# Gut microbiome of the grape berry moth, Paralobesia viteana (Lepidoptera: Tortricidae) larvae through the grape ripening process revealed by high-throughput 16S and 18S rRNA sequencing

**DOI:** 10.1099/mgen.0.001756

**Published:** 2026-06-17

**Authors:** Laura Laiton, Flor E. Acevedo

**Affiliations:** 1 Department of Entomology, Pennsylvania State University, University Park, PA, 16802, USA

**Keywords:** amplicon metagenomic sequencing, bacterial microbiota, fruit-feeding insect, fungal microbiota, *Vitis*

## Abstract

The grape berry moth (GBM) *Paralobesia viteana* (Lepidoptera: Tortricidae) is an important pest of grapes in eastern North America. The larvae damage grape clusters by direct feeding and by increasing susceptibility to fungal and bacterial pathogens. In this study, we sequenced the V3–V4 region of the 16S rRNA gene and the V4 region of the 18S rRNA gene to characterize the composition and diversity of GBM larval gut bacterial and fungal communities when fed on immature and mature ‘Concord’ grapes. The data were analysed with QIIME 2, and downstream analyses included taxonomic composition, differential abundance, phylogenetic, functional and alpha/beta diversity analyses. While overall bacterial community diversity did not differ significantly between treatments, differential abundance analysis identified specific bacterial taxa enriched in each larval group. Ninety-three per cent of the bacterial communities belonged to the phylum *Proteobacteria*, and some may play roles in amino acid and carbohydrate metabolism in the insect gut. Analyses of the 18S rRNA region showed significant taxon-level compositional differences in fungal communities between larvae grown on grapes at different ripening stages. *Ascomycota* was the dominant phylum (98%) present in the guts of larvae fed on mature grapes, while larvae fed on immature grapes mainly contained fungi within the *Cryptomycota* (51%). Larvae fed on ripe grapes had a 10-fold higher fungal abundance and were enriched in Saccharomycetales yeasts. Several of the identified microbial taxa in larval guts are commonly found in grapes, which suggests they might be transient insect residents that are ingested with the diet. In conclusion, diet strongly shaped GBM gut-associated fungal communities; specific bacterial taxa also differed between larval groups despite similar overall bacterial diversity. These results contribute to basic knowledge of gut-associated microbes in fruit-feeding insects.

**Impact Statement**

This study provides the first comprehensive characterization of the gut microbiome in grape berry moth (*Paralobesia viteana*) larvae, addressing a critical knowledge gap in fruit-feeding lepidopteran ecology. The work reveals that while bacterial communities remain stable across grape ripening stages and are dominated by *Proteobacteria* (93%), differential abundance analysis identified specific bacterial taxa that differed significantly between larval groups. Fungal communities differ markedly, with a 10-fold higher abundance and enrichment of Saccharomycetales yeasts in larvae fed on mature grapes compared to those fed on immature fruit. These findings advance understanding of diet-dependent microbial community assembly in frugivore insects and provide foundational data for future studies on insect adaptation to sugar-rich but nutrient-poor diets, while establishing methodological frameworks for comparative microbiome studies in tortricid fruit borers.

## Data Summary

The raw sequence data are accessible at the National Center for Biotechnology Information (NCBI) Sequence Read Archive database under the BioProject number PRJNA952655 and the accessions listed in Table S8. The code for the metagenomic analyses is available at https://github.com/lauralaiton/GBM_metagenomics.

## Introduction

Insects, the most diverse group of organisms on the planet, have evolved strong associations with symbionts. Micro-organisms from all domains, including bacteria, fungi, archaea, protozoans and viruses, live in insect bodies, with bacteria being the most prevalent [1]. Most insects have specialized gut microbes that provide a diverse range of benefits, including environmental adaptation, nutrient acquisition, pathogen resistance, detoxification of xenobiotics and enhancement of immunity and fitness [1,4]. In some insect species, their associated microbial symbionts play critical nutritional roles and contribute to host health [5,7]. For instance, the wood-feeding termite (*Reticulitermes chinensis*) and the ship timber beetle (*Elateroides flabellicornis*) rely on gut-associated yeast and other fungal communities to digest recalcitrant wood compounds [8,9]. Gut-associated bacteria of the olive fly larvae (*Bactrocera oleae*) support higher egg production when fed on diets with low nitrogen content [10]. Moreover, research in tephritid fruit flies emphasizes the substantial role of gut microbiota in modulating host immune responses, pathogen resistance and overall fitness, making the interaction between microbial communities and host immunity a promising research area with significant implications for pest control [11].

The microbiome composition of insects is shaped by multiple exogenous and endogenous factors such as diet type, life stage, environment, sex and phylogeny. Host diet is the primary factor altering the gut microbiome community structure of various insect groups, including lepidopterans [3], termites [12], bees [13], dragonflies [14], beetles [15,16] and *Drosophila melanogaster* [17], among others. This could be explained by two interacting factors: first, some microbial communities may be transient residents ingested with the diet [3,18], and second, consuming certain phytochemicals may have greater detrimental impacts on some microbes or may stimulate growth in others [19]. Conversely, in some insect groups, the composition of the gut microbiome community may be unaffected by dietary changes and is closely associated with host phylogeny. Some examples include cockroaches [20], crickets [21], butterflies [18,22] and sap-sucking insects [23].

Lepidopterans (butterflies and moths) constitute the second largest order of insects, comprising ~180,000 species in 126 families [24]. Most of them are phytophagous, and some are important agricultural pests. Lepidoptera larvae feed on different plant tissues, including flowers, fruits, seeds, leaves, stems and roots [25]. Despite the economic and ecological importance of lepidopterans, our knowledge of their microbiota composition is limited to less than 0.1% of the identified species [2,3]. Nonetheless, a comprehensive review of lepidopteran gut communities highlights progress in characterizing both stable and transient microbial associations shaped by ecological context [3]. To date, most of this work has focused on leaf-feeding caterpillars, with comparatively few studies addressing the microbiota composition in species with alternative diets. For instance, the gut microbiota of fruit-feeding lepidopterans remains poorly characterized [26,27], except for a few species. The oriental fruit moth, *Grapholita molesta* (Lepidoptera: Tortricidae), hosts a microbiome in which *Proteobacteria* and *Firmicutes* predominate across all developmental stages [28]. A comparative study of two fruit-feeding pests, the peach fruit moth, *Carposina sasakii* (Lepidoptera: Carposinidae) and *G. molesta*, further revealed a shared core microbial genera (*Pseudomonas*, *Gluconobacter*, *Acetobacter* and *Pantoea*), suggesting these taxa may play conserved roles in fruit digestion and nutrient acquisition among lepidopteran frugivores [29].

The grape berry moth (GBM) (*Paralobesia viteana*) (Lepidoptera: Tortricidae) is a pest native to the eastern USA that completes its development exclusively on wild and cultivated grape clusters (*Vitis* spp.) [30]. The larvae of GBM damage grape clusters directly by feeding on berries throughout the growing season and indirectly by predisposing damaged clusters to pathogen attack [31,32]. GBM completes its life cycle in about 32 days at 25 °C, and 3–5 generations can develop yearly in the northeast USA, depending on temperature [33,34]. Adults emerge in spring from overwintering pupae, and females lay eggs on flower clusters or grape berries at any developmental stage. Upon emergence, young larvae burrow into the grapes and feed on the pulp. The GBM larvae undergo four instar stages in about 18.5 days at 25 °C and can damage multiple grapes within the same cluster [34]. Later generations are especially harmful, as larval feeding promotes secondary infection with pathogens that decompose the fruit. All grape cultivars are susceptible to GBM from bloom to harvest, despite major changes in chemistry during berry development. Unripe grapes have lower sugar content, lower pH, higher levels of malic acid and lower levels of phosphoric acid compared with mature grapes [35]. After veraison (the onset of fruit ripening), grapes accumulate high concentrations of sugars (glucose, fructose and sucrose) and phenolic compounds, especially in red cultivars [35]. These nutritional and chemical changes in fruit composition may affect the GBM larval microbiome and subsequently influence its fitness, although this relationship remains to be studied. GBM is a good model to study the gut microbiome of fruit-borer lepidopterans because it develops primarily on grape clusters at any ripening stage.

In this study, we investigated the composition and diversity of gut-associated bacteria and fungi in *P. viteana* larvae feeding on grape berries at different ripening stages (unripe and ripe) using the 16S and 18S rRNA genes high-throughput sequencing approach. We hypothesize that the insect would have a lower abundance and diversity of microbes when fed on unripe grapes compared with those fed on ripe berries. Our results contribute to current knowledge of gut-associated symbionts in lepidopteran fruit borers and improve current understanding of grape berry moth–microbe–plant interactions.

## Methods

### Sample collection and processing

Grape berry moth-infested berries of the ‘Concord’ cultivar (*Vitis labrusca* L. or *Vitis × labruscana* L.H. Bailey) were collected before the onset of ripening (pre-veraison) in July 2021 (average fresh berry weight=2.48 g, pH 2.57 and 3.39° Brix) and at the ripening stage in September 2021 (average fresh berry weight=3.53 g, pH=3.19 and 10.91 degrees Brix). The samples were collected from a 76-year-old vineyard (42° 12′ 23.4″ N 79° 53′ 49.9″ W) in North East (PA, USA). Grape berries were collected in clean plastic containers, placed in a cooler and transported to the lab at Penn State Behrend (Erie, PA 16563). GBM-infested berries were immediately dissected under a stereomicroscope (Leica S9D Nuhsbaum Inc, McHenry, IL, USA) to collect actively feeding larvae in their last instar. Each larva was washed with distilled water, surface sterilized with 70% ethanol for 30 s and rinsed with sterile distilled water. Whole guts with gut contents were individually extracted from GBM larvae pinned on sterile sylgard-coated petri dishes under a stereomicroscope (Leica S9D). The larvae were immobilized by pinning the anterior end (head) and the posterior end onto the dissecting dish with two sterile entomological needles; two incisions were made with sterile dissecting scissors: one in the posterior part between the first pair of legs and the head capsule to separate the foregut from the head and the other in the posterior end of the larva to expose the hindgut.

The exposed end of the hindgut was carefully dragged with sterile forceps until the whole gut was extracted from the insect. Lastly, the gut was rinsed in sterile 1X PBS buffer before being placed in a 1.5-ml Eppendorf tube. Each replicate comprised a pooled sample of ten guts. Samples were stored at −80 °C for further DNA purification. Four and three replicates of larvae fed on immature (pre-veraison) and mature (ripe) grapes, respectively, were used for 16S rRNA Illumina sequencing. For 18S rRNA, we used three and four replicates of larvae from immature and mature grapes, respectively.

### DNA extraction and quantification

Total microbial DNA was purified using the EZNA Fungal DNA Mini Kit (Omega Bio-Tek, Darmstadt, Germany) following the manufacturer’s protocol. The DNA was then quantified using a NanoDrop^™^ One^c^ spectrophotometer (Thermo Scientific, USA) and stored at −20 °C for further use.

### PCR amplification

The genomic DNA extracted from the samples was used for amplifying the variable region V3–V4 of the 16S rRNA gene of bacteria and archaea and the V4 region of the 18S rRNA gene of fungi. Amplicons were constructed using the primer sets for the 16S rRNA gene: forward 341F (5′-CCT AYG GGR BGC ASC AG-3′) and reverse 806R (5′-GGA CTA CNN GGG TAT CTA AT-3′) according to Klindworth *et al*. [36]. The primer sets for amplifying the 18S rRNA gene were forward 528F (5′-GCG GTA ATT CCA GCT CC AA-3′) and reverse 706R (5′-AAT CCR AGA ATT TCA CCT CT-3′) based on Banos *et al*. [37]. A PCR mixture (20 µl per reaction) was prepared with 0.6 U of Phusion^™^ High-Fidelity DNA Taq Polymerase (New England BioLabs, Ipswich, USA), 0.5 µM of each forward and reverse primer, 200 µM of dNTPs, 1X Phusion HR reaction buffer and 50 ng of template DNA. The PCR cycle for the 16S V3–V4 region had an initial denaturation step of 98 °C for 2 min, followed by 30 cycles at 98 °C for 10 s, annealing at 50.5 °C for 30 s and extension at 72 °C for 30 s with a final extension step at 72 °C for 7 min. The 18S gene PCR cycle had a denaturation of 95 °C for 3 min, followed by 34 cycles at 95 °C for 30 s, annealing at 50 °C for 30 s and extension at 68 °C for 1 min with a final extension step at 68 °C for 7 min. The PCR products were visualized in 2% TAE agarose gel electrophoresis stained with Gel Red^®^ (Biotium, San Francisco, CA, USA) in a GelDoc Go imaging system (Bio-Rad, Hercules, California, USA). The selected primer sets (341F/806R for 16S V3–V4 and 528F/706R for 18S V4) are widely used for profiling insect-associated microbiomes; however, short amplicon sequencing provides limited species-level resolution. In particular, the 18S V4 region exhibits strong taxonomic bias and limited discriminatory power for fungi, while the 16S V3–V4 region provides only moderate species-level resolution for bacteria. Accordingly, species-level annotations derived from amplicon sequence variants (ASVs) were not included in the manuscript. No mock community was included, and classification accuracy was interpreted conservatively based on established pipelines and reference databases.

### Library preparation and 16S/18S rRNA sequencing

Libraries for Illumina sequencing were generated using the NEBNext^®^ Ultra^™^ DNA Library Preparation Kit (New England BioLabs, Ipswich, MA, USA) according to the manufacturer’s instructions, with the addition of index codes. The quality of the libraries was assessed using the Qubit@ 2.0 Fluorometer (Thermo Scientific) and the Agilent Bioanalyzer 2100 system. The 250 bp paired-end libraries were sequenced using an Illumina NovaSeq 6000 platform (Illumina, San Diego, CA, USA) at Novogene Corporation Inc. (Beijing, China).

### 16S/18S rRNA bioinformatics and data analysis

The raw paired-end 16S and 18S rRNA sequences were processed and analysed using the Quantitative Insights Into Microbial Ecology (QIIME) 2 software package version 2022.8 (https://qiime2.org: [38]). Demultiplexed FASTQ files were imported into QIIME 2 with the *qiime tools import* input-format ‘PairedEndFastqManifestPhred33V2’ command. The q2-dada2 plugin was used for filtering chimeric sequences, denoising, trimming and clustering. The 16S rRNA forward and reverse sequences were trimmed to 225 bp and 223 bp, respectively, whereas the 18S rRNA sequences were trimmed to 218 bp forward and reverse. The quality-filtered trimmed sequences were used to create the ASV tables, also called feature tables [39]. The feature naïve-Bayesian classifier was trained with the *qiime feature-classifier* fit-classifier-naive-bayes tool using the Greengenes database version 13_8 clustered at 99% sequence identity (99% Operational Taxonomic Units [OTUs]) and the silva database release 138 clustered at 99% sequence identity (99% OTUs) for 16S and 18S sequences, respectively. Subsequently, the reference reads were extracted from the reference databases using the *qiime feature-classifier extract-reads* command, which is based on matches to the corresponding 341F/806R or the 528F/706R primer pairs.

The taxonomic classification and clustering of the ASVs were done with the *feature-classifier* plugin using default settings [40]. Features were filtered out based on taxonomy using the *qiime taxa filter-seqs* command to remove possible contaminants, such as chloroplasts and mitochondria in the 16S features, and Arthropoda, Nematozoa, dinoflagellate and 45 other phyla in 18S features. A phylogenetic tree of the filtered ASVs was generated using the *qiime phylogeny align-to-tree-mafft-fasttree* plugin. The alpha and beta diversity metrics of bacterial communities were calculated with the *qiime diversity core-metrics-phylogenetic* plugin based on normalized ASV abundance data, which was obtained using the sample containing the smallest set of feature sequences as sampling depth to avoid heterogeneity. The obtained data were then exported in BIOM format for downstream analyses. For fungi, diversity metrics were not carried out due to pronounced differences in the number of ASVs between treatment groups that could not be normalized in *qiime* without losing a large number of samples. As a result, treatment comparisons for fungi were descriptive.

Taxonomic abundance plots of bacterial and fungal features were built with the ggplot2 package in R (version 4.2.0). Shared features across both larval groups were visualized by Venn diagrams built with Matplotlib Pyplot in Python (version 3.10.2). The phylogenetic trees of ASVs were visualized with the EMPress-QIIME 2 viewer. Heat maps illustrating the most abundant gut microbial communities at the family and genus levels were built using the qiime2r package in R.

The variability of bacterial ASVs within samples was calculated using alpha diversity indexes. The alpha diversity metrics calculated for bacterial communities included observed features (accounts for the number of taxonomic groups), Shannon diversity (a measure of richness), Pielou’s evenness (distribution of ASV abundances within a sample), Chao1 (an estimator of ASV richness), Fisher (describes the relationship between the number of ASVs and their abundances) and Simpson (accounts for dominance and diversity within the ASV community). Statistical differences in gut-associated microbial composition between GBM larvae fed on immature and mature grapes were assessed using Analysis of Composition of Microbiomes with Bias Correction (ANCOM-BC) implemented in QIIME 2 [41]. Feature tables were collapsed to the order, family and genus levels, and low-frequency taxa were filtered prior to their analysis. Alpha diversity data were exported, and the results were visualized in R using the ggplot2 package.

The variability in the composition of bacterial communities between groups was calculated using beta diversity indexes. To visualize clustering patterns of bacterial communities between larvae fed on immature and mature grapes, the beta diversity data were analysed with principal coordinate analysis (PCoA) and non-metric multidimensional scaling (NMDS) plots, which were constructed based on phylotype abundances (Bray–Curtis distance). Beta diversity plots were built using MicrobiomeAnalyst (https://www.microbiomeanalyst.ca/). To determine statistical differences in community structure at the ASV level, both groups of larvae were compared using the permutational multivariate ANOVA (PERMANOVA) using the unweighted UniFrac distance (presence or absence of observed ASVs), weighted UniFrac distance (relative abundance of ASVs) and Bray–Curtis dissimilarity analysis (differences in ASV composition between sample groups) using QIIME 2.

To predict the functional metabolic pathways associated with the bacterial ASVs between larval groups, we used the Phylogenetic Investigation of Communities by Reconstruction of Unobserved States (PICRUSt) [42] based on the Kyoto Encyclopedia of Genes and Genomes (KEGG) orthologue database [43]. The KEGG identifiers were classified at level 3 (i.e. assigned to specific pathway maps, the most detailed layer of the KEGG hierarchy). Functional differences between groups were tested using Welch’s t-test with the Benjamini–Hochberg false discovery rate (FDR) correction. Dissimilarities across the relative frequencies of taxa were illustrated with a PCA plot using the Bray–Curtis distance matrix. Figures, including a cluster heat map of the metabolic pathway abundance table, were generated using the Statistical Analysis of Taxonomic and Functional Profiles software [44]. A workflow and required pipelines describing the implementation of our QIIME 2 analysis are publicly available in GitHub (https://github.com/lauralaiton/GBM_metagenomics).

## Results

### GBM gut-associated bacteria

We obtained 1,266,163 assembled Illumina paired-end sequences for the V3–V4 regions of the 16S rRNA gene from larval guts. The average library size was 180,880 (± 6,858 sd) sequences per replicate. After trimming and quality control, we obtained 466,516 and 396,619 bacterial sequences from larvae fed on immature and mature grapes, respectively (Table S1, available in the online Supplementary Material).

**Fig. 1.**
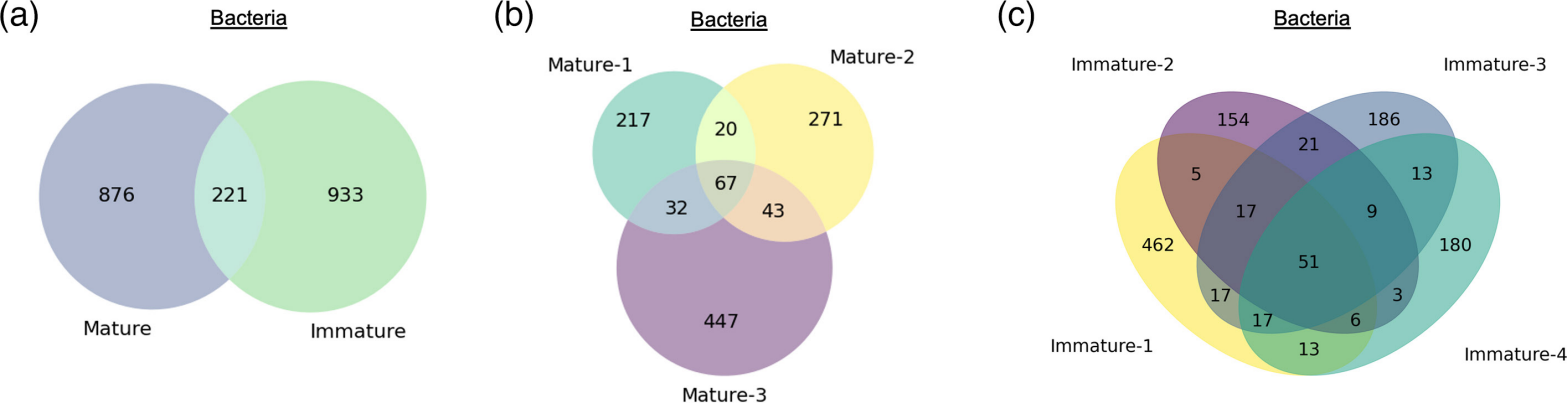
Venn diagrams of *P. viteana* gut bacterial communities at the ASV feature level. (**a**) Features of larval groups fed on mature or immature grapes. (**b**) Features of larvae fed on mature grapes (mature group). (**c**) Features of larvae fed on immature grapes (immature group).

In total, 3569 ASVs were identified using QIIME 2 at 97% identity. After taxonomic classification, 1571 bacterial taxonomic units (ASVs) were filtered due to low ASV sequence counts and contaminant matchings. From these, 1,998 ASVs were used in comparative analyses between the two larval groups (Fig. S1). Overall, 221 features were found in both larval treatment groups, i.e. those fed on immature and mature grapes (Fig. 1a). The number of shared features across samples was 67 and 51 for larvae fed on mature and immature grapes, respectively (Fig. 1b, c).

### Taxonomic classification of GBM gut-associated bacterial communities

**Fig. 2.**
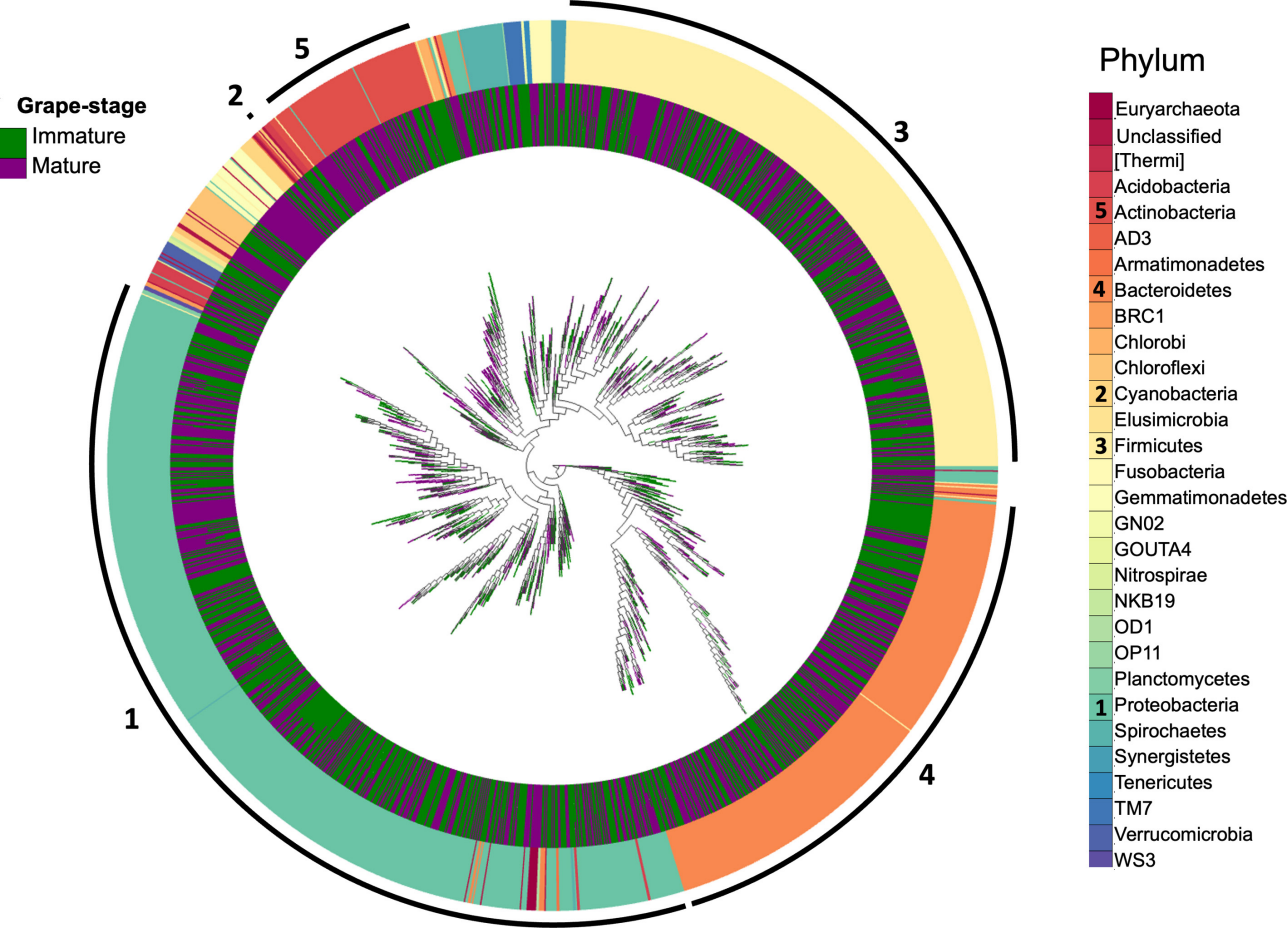
Phylogenetic tree of bacterial ASVs generated from 16S rRNA gene sequencing data of *P. viteana* larval guts fed on immature or mature grapes. The phylogenetic tree is shown at the ASV feature level. The phylogenetic tree depicts relationships among ASVs (features), and branch colours indicate the treatment group (guts from larvae fed mature or immature grapes) in which each ASV was detected. The inner concentric ring indicates the grape stage. The outer ring represents taxonomic assignment at the phylum level, with colours corresponding to the legend. The numbers 1 to 5 represent the top five most abundant phyla across all samples.

Taxonomic assignments for ASVs were distributed across 29 bacterial phyla, 74 classes, 109 orders, 162 families and 243 genera. The taxonomic profiling of the metagenome indicated that the gut microbiota in both larval groups and across samples was dominated by *Proteobacteria* (93.4%), followed by *Cyanobacteria* (3.07%), and to a lesser extent *Firmicutes* (1.87%) and *Bacteroidetes* (1.09%) (Table 1 and Fig. S2A). The phylogenetic relationships among taxa are illustrated in Fig. 2. Taxa from larvae fed on mature grapes were broadly distributed across the major phyla, whereas taxa from larvae fed on immature grapes appeared more concentrated within specific phylogenetic clades. *Proteobacteria* formed the largest phylogenetic sector and were bordered by several smaller phylum-level lineages. The clearest neighbouring major sector was *Bacteroidetes*, while *Firmicutes* and *Cyanobacteria* occupied separate, more distant regions of the tree. *Cyanobacteria* were positioned near the transition between *Actinobacteria*- and *Firmicutes*-associated regions, supporting their phylogenetic separation from the *Proteobacteria*/*Bacteroidetes* assemblages. Seventy-four classes were identified (Fig. S2B), of which 11 were found in all samples, including the most abundant *Alphaproteobacteria* (84.02%), *Gammaproteobacteria* (11.17%) and *Clostridia* (1.6%) (Table 1). The most abundant orders in all samples were *Rickettsiales* (82.5%), *Enterobacteriales* (9.65%) and *Rhizobiales* (1.84%) (Table 1) from the 109 orders identified (Fig. S2C).

**Table 1.** Relative abundance (RA, %) of gut bacterial and fungal communities across taxonomic levels in *P. viteana* larval guts fed on immature and mature grapes. The table presents the mean relative abundance of the top 10 bacterial and fungal taxa identified across all samples at the phylum, class and order levels

Bacteria						Fungi					
Phylum		Class		Order		Phylum		Class		Order	
Taxon	**RA (%)**	Taxon	**RA (%)**	Taxon	**RA (%)**	Taxon	**RA (%)**	Taxon	**RA (%)**	Taxon	RA (%)
*Proteobacteria*	93.38	*Alphaproteobacteria*	84.02	*Rickettsiales*	82.5	*Ascomycota*	97.67	*Saccharomycetes*	82.98	*Saccharomycetales*	83.24
*Cyanobacteria*	3.07	*Gammaproteobacteria*	11.17	*Enterobacteriales*	9.65	*Cryptomycota*	1.66	*Leotiomycetes*	12.91	*Helotiales*	12.94
*Firmicutes*	1.87	*Clostridia*	1.6	*Rhizobiales*	1.84	*Basidiomycota*	0.47	*LKM11*	1.35	*LKM11*	1.36
*Bacteroidetes*	1.09	*Betaproteobacteria*	1.14	*Clostridiales*	1.63	*Chytridiomycota*	0.16	*Dothideomycetes*	1.15	*Hypocreales*	0.45
*Actinobacteria*	0.35	*Bacteroidia*	0.82	*Burkholderiales*	1.1	*Aphelidea*	0.02	*Sordariomycetes*	0.69	c_;Dothideomycetes;o_Unclassified	0.43
*Synergistetes*	0.07	*Deltaproteobacteria*	0.36	*Xanthomonadales*	0.98	*Mucoromycota*	0.01	*Eurotiomycetes*	0.32	*Capnodiales*	0.41
*Fusobacteria*	0.07	*Bacilli*	0.33	*Rhodospirillales*	0.85	*Incertae_Sedis*	0.01	*Exobasidiomycetes*	0.19	c_Cryptomycota;o_Incertae_Sedis	0.32
*Spirochaetes*	0.03	*Actinobacteria*	0.28	*Bacteroidales*	0.84	*LKM15*	0	*Chytridiomycetes*	0.16	*Pleosporales*	0.32
*Chloroflexi*	0.03	*Sphingobacteriia*	0.13	*Pseudomonadales*	0.49			*Incertae_Sedis*	0.11	*Eurotiales*	0.32
*Gemmatimonadetes*	0.02	*Flavobacteriia*	0.11	Others	0.11			*Microbotryomycetes*	0.11	*Entylomatales*	0.19
Others	0	Others	0.03					Others	0.02	Others	0.04

**Fig. 3.**
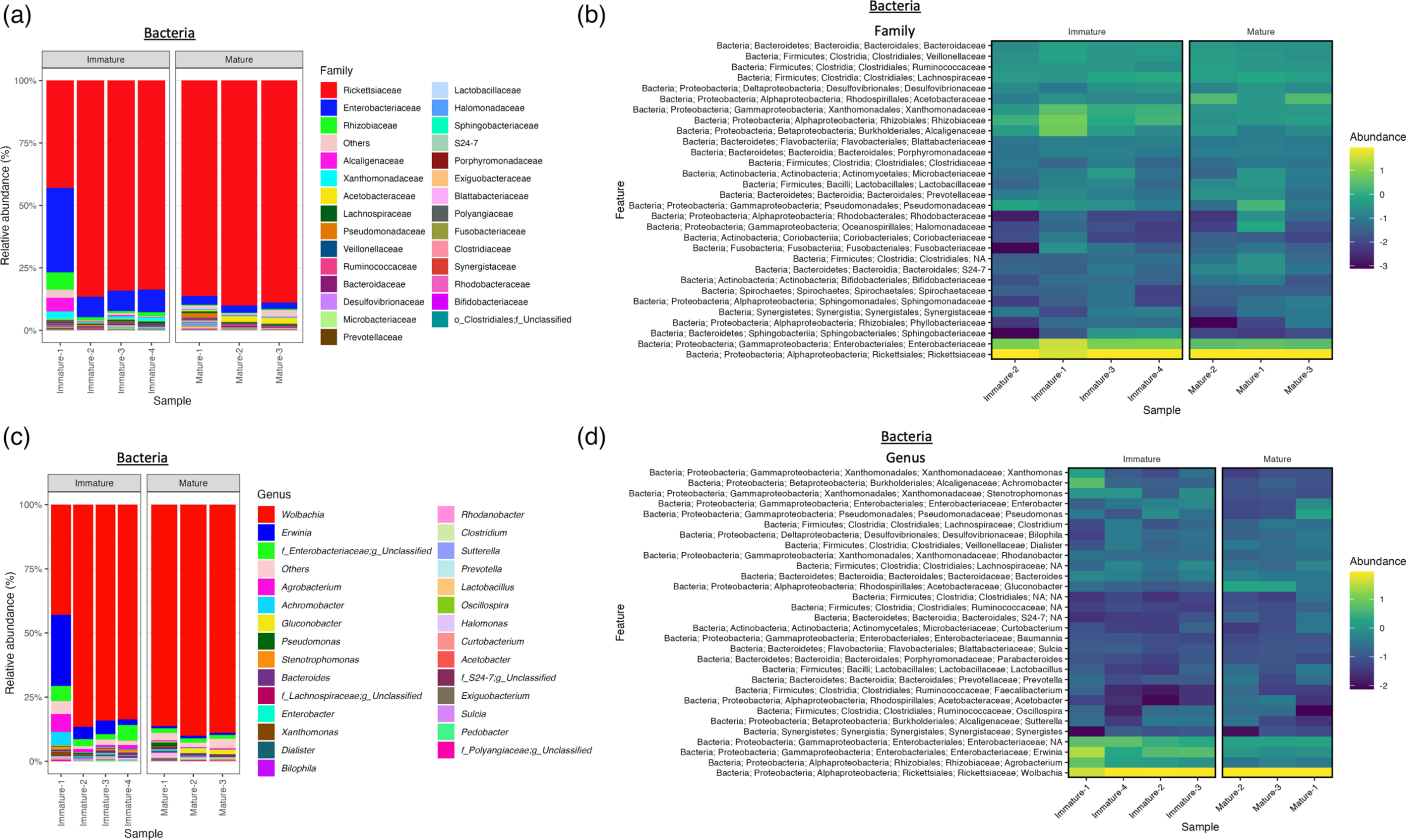
Relative abundance and heat maps of the gut bacterial communities of *P. viteana* larvae fed on immature and mature grapes at the (**a, b**) family and (**c, d**) genus levels. (**a, c**) Relative abundance (%) of gut bacterial communities across all samples. Taxa below the top 30-abundance cut-off are represented as ‘Others’. ‘Unclassified’ represents taxa that were not assigned to any group. (**b, d**) Heat maps illustrating the 30 most abundant gut bacterial communities across samples; colours represent log-transformed relative abundances, where yellow indicates higher and purple indicates lower abundance values.

A total of 162 families were identified in the samples. The distribution of relative abundances at the family level is shown in Fig. 3a. Overall, *Rickettsiaceae* was the most representative family (Fig. 3a, b), accounting for more than 77% of all samples’ abundance, except for the immature-1 sample, which represented 42% of the total abundance (Fig. 3a). *Enterobacteriaceae* was the next most represented family across all samples (Fig. 3a, b), especially in the immature-1 sample with an abundance of 33.7% (Fig. 3a). The remaining families had abundances lower than 7% across samples (Fig. 3a). Differential abundance analysis using ANCOM-BC identified five bacterial families that differed significantly between larvae fed on immature and mature grapes: *Leptotrichiaceae*, *Acetobacteraceae*, *Paraprevotellaceae*, the unclassified *Bacteroidales* and *Rhizobiaceae*. The *Bacteroidales* lineage was significantly enriched in larvae fed on mature grapes, whereas the *Rhizobiaceae* lineage was enriched in larvae fed on immature grapes (*q*<0.05, Fig. 3b and Table S2).

Most reads from GBM fed on immature and mature grapes were taxonomically classified down to the genus level, and the distribution of relative abundances is shown in Fig. 3c. From the 243 genera found, *Wolbachia* was the most predominant genus across all samples, followed by *Erwinia*, Agrobacterium and a non-identified genus belonging to the Enterobacteriaceae family (Fig. 3c, d). Differential abundance analysis identified ten genera that varied significantly between larvae fed on immature and mature grapes: *Leptotrichia*, *Gluconacetobacter*, *Gluconobacter*, *Neisseria*, *Prevotella*, *CF231*, *Peptostreptococcus*, an unclassified *Bacteroidales* lineage, *Agrobacterium* and an unclassified genus within the *Christensenellaceae*. The unclassified *Bacteroidales* lineage was enriched in larvae fed on mature grapes, whereas *Agrobacterium* and the unclassified genus within the *Christensenellaceae* were enriched in larvae fed on immature grapes (*q*<0.05, Table S3).

Microbiome analysis in insects often involves removing *Wolbachia*-associated sequences when they are found in 16S rRNA libraries to reduce bias [45]. In this study, the overall relative abundances at both the phylum and genus levels remained similar after removing the *Wolbachia* sequences. The list of the top ten most abundant phyla and genera did not change after removing *Wolbachia*, and the microbiota was still dominated by the phylum *Proteobacteria* and the genus *Erwinia* (Fig. S3).

### Diversity analysis

**Fig. 4.**
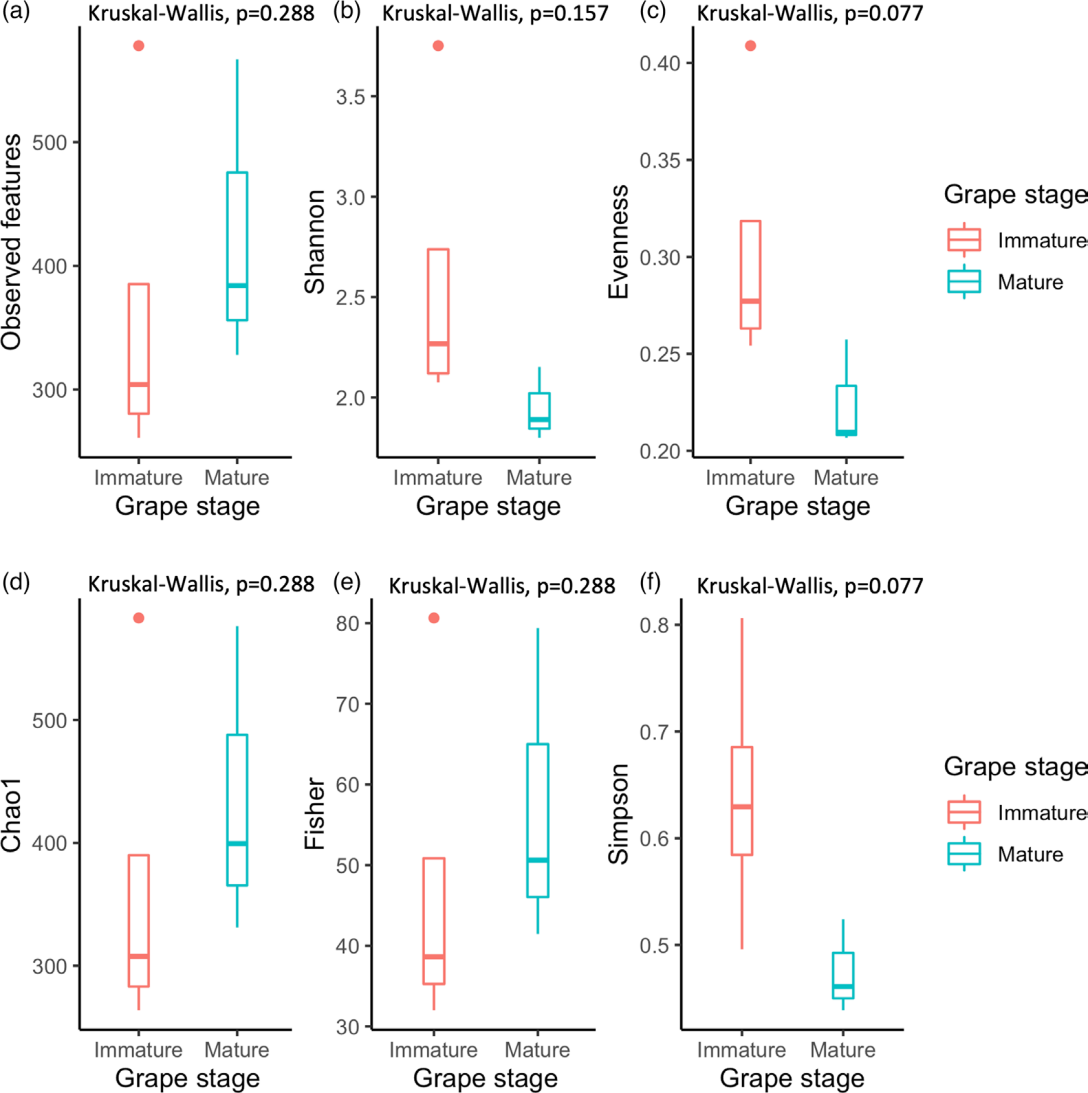
Alpha diversity analyses of *P. viteana* larval gut bacterial communities using the (**a**) number of observed features, (**b**) Shannon, (**c**) Pielou’s evenness, (**d**) Chao1, (**e**) Fisher and (**f**) Simpson indexes.

The rarefaction curve reached a plateau, suggesting that the gut-associated microbial communities were sampled exhaustively and captured the majority of ASVs (Fig. S4); therefore, the sampling conducted was appropriate for diversity analyses. The alpha diversity within each larval group was analysed using the observed features, Shannon, Pielou’s evenness, Chao1, Fisher and Simpson indexes (Fig. 4). The observed features ranged from 261 to 578 across all samples. Similarly, Chao1 ranged from 264 to 583; however, no significant differences were found in either of these indices between larvae groups (Kruskal–Wallis test: H=1.13, *P=*0.288 and H=1.13, *P=*0.288, respectively). The Shannon index ranged from 1.79 to 3.75, and the Pielou’s evenness index ranged from 0.408 to 0.206; these two indices were not significantly different between larvae fed on immature or mature grapes (Kruskal–Wallis test: H=2.0, *P=*0.157 and H=3.13, *P=*0.077, respectively). Similarly, the Fisher index, which ranged from 80.63 to 32.03, and the Simpson index, which ranged from 0.806 to 0.439, were not significantly different between larval groups (Kruskal–Wallis test: H=1.13, *P=*0.288 and H=2.0, *P=*0.157, respectively).

**Fig. 5.**
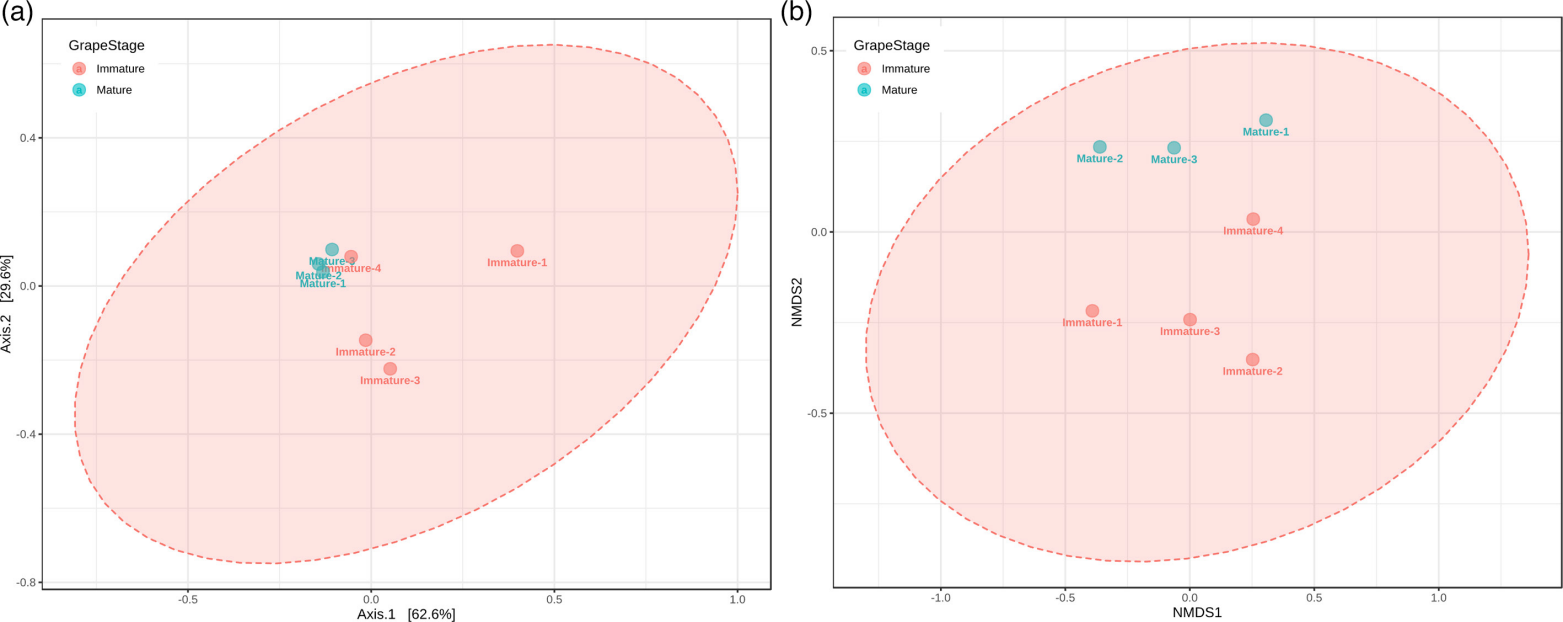
Beta diversity comparison of *P. viteana* larval gut bacterial communities at the feature level when fed on immature and mature grapes. (**a**) PCoA, (**b**) NMDS. Both plots are based on the Bray–Curtis distance matrix. Dots in panels represent each sample for the grape stages.

The distribution of GBM larval gut bacterial communities did not show clear separation between larvae fed on immature and mature grapes according to the PCoA and NMDS plots performed at the feature level (Fig. 5). The sample ‘Immature-4’ tends to overlap with the samples of the mature group. Consequently, PERMANOVA analysis showed that the beta diversity metrics of unweighted UniFrac, weighted UniFrac and Bray–Curtis were not significantly different between the two larval groups (Table 2).

**Table 2.** PERMANOVA results for three beta diversity metrics comparing the effect of GBM larvae diet on gut bacterial composition

Dissimilarity index	Permutations	Pseudo-F	***P*-value**
Unweighted UniFrac	999	1.047	0.315
Weighted UniFrac	999	1.629	0.151
Bray–Curtis	999	1.629	0.166

After removing *Wolbachia* sequences from all samples, the similarities in alpha diversity indices between larvae fed on immature and mature grapes did not change (Table S4) (observed ASV features: *H*=1.99, *P=*0.157; Shannon: *H*=1.99, *P=*0.157; Pielou’s evenness: *H*=1.99, *P=*0.157; Chao1: *H*=1.99, *P=*0.157; Fisher: *H*=1.13, *P=*0.289; Simpson: *H*=0.49, *P=*0.479). Furthermore, the microbial community structure of larvae fed on different grape ripening stages also remained unchanged (Bray–Curtis distance: pseudo-*F*=2.491; *P*=0.115; Weighted-UniFrac: pseudo-*F*=2.491; *P*=0.119; unweighted-UniFrac: pseudo-*F*=1.127; *P*=0.193).

### Functional prediction of GBM bacterial microbiota

**Fig. 6.**
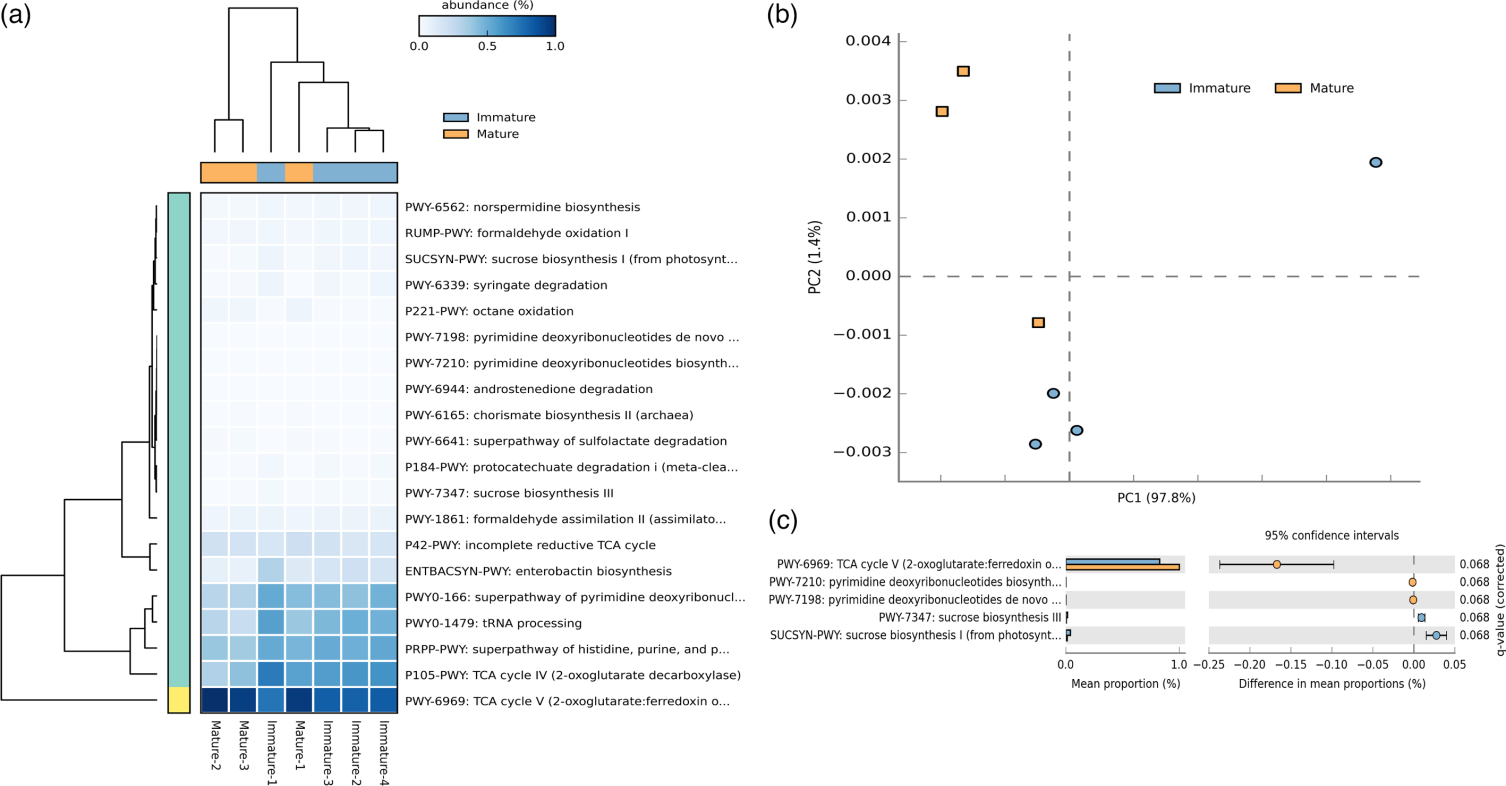
Common predicted functional metabolic KEGG pathways of *P. viteana* gut bacteria (level 3). (**a**) Heat maps and hierarchical clustering of the pathways. The top 20 pathways were listed, and colours shift from white (lower) to dark blue (higher) according to the pathway abundance in each sample, indicated by the logarithmic values of the relative abundances. (**b**) PCA plot of different functional pathways between larvae fed on immature and mature grapes. (**c**) Extended bar plot of different functional pathways between immature and mature larval groups. Columns indicate the mean proportion (%) of different MetaCyc pathways predicted by PICRUSt2. Differences between groups are shown with 95% confidence intervals. Since no significant differences were found, the five pathways with the lowest *p*-values (corrected for Storey FDR using Welch’s t-test) are shown.

The abundances of KEGG pathways were predicted by PICRUSt2 based on 16S rRNA gene sequences, and their abundances were displayed in a heatmap (Fig. 6a). The roles of GBM gut-associated bacteria mostly comprised the metabolism of carbohydrates, amino acids, polyphenols, sulphur and methylotrophic compounds. Other functions enriched in both larval groups included processing of genetic information (replication, translation and repair), degradation of aromatic and organic compounds and amino acid biosynthesis. Despite the output of the PCA plot (Fig. 6b), there were no significant differences in functional pathways between the two larval groups (Welch’s t-test, Storey FDR *P*>0.05) (Fig. 6c).

### GBM gut-associated fungi

High-throughput 18S rRNA sequencing analysis yielded a total of 1,225,918 assembled sequences for the V4 region from the 7 samples of GBM larval guts. The library’s size was 175,131±4350 (sd) raw tags on average per replicate. After quality control, we obtained 452,949 and 621,117 effective tags for immature and mature samples, respectively (Table S5). From these sequences, 800 ASVs were identified at 70% identity. Due to the lack of specificity of the 18S rRNA region, the taxonomic filter dropped 48 contaminant ASVs at the phylum level belonging to non-fungal micro-organisms. A total of 156 unique ASVs were conserved and used in comparative analyses between the 2 larval groups (Fig. S5A). However, due to marked differences between the two larval groups, the number of sequences per sample could not be normalized to carry out alpha and beta diversity analyses of the microbiota. All samples from larvae fed on immature grapes presented very low ASV frequencies compared to those fed on mature grapes (Fig. S5B). To proceed with diversity analyses, conserving all the immature samples, we could have chosen a sampling depth of 1,242; however, this would only have allowed us to retain 4% of our total features. Therefore, diversity analyses of fungi in GBM gut-associated communities were not carried out, and diversity data were analysed descriptively.

**Fig. 7.**
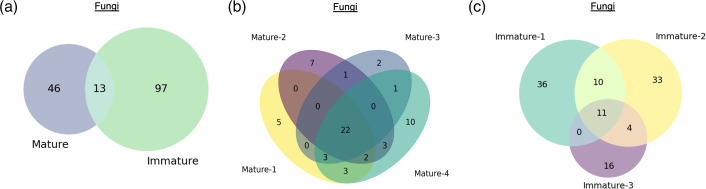
Venn diagram of *P. viteana* larval gut fungal communities at the ASV feature level. (**a**) Features of both larval groups. (**b**) Features of larvae fed on mature grapes (mature group). (**c**) Features of larvae fed on immature grapes (immature group).

From the 156 conserved ASVs, 13 were found in both groups of larvae (Fig. 7a). Samples from larvae fed on immature grapes (labelled as ‘immature’) had almost twice as many unique ASVs as those fed on mature grapes. All samples from the mature group shared 22 ASVs (37.3%) (Fig. 7b), and those from the immature group shared 11 ASVs (10%) (Fig. 7c).

### Taxonomic classification of GBM gut-associated fungi communities

Detected fungi and yeasts were classified into 9 phyla, 19 classes, 32 orders, 43 families and 56 genera. Those sequences that were not assigned to known microbial taxa were designated as ‘unclassified’. *Ascomycota* was the most dominant phylum across the larval group fed on mature grapes, representing 97.67% of relative abundance (Fig. S2E and Table 1), whereas the larvae fed on immature grapes contained mainly taxa in the *Cryptomycota* (51%) and *Ascomycota* (34.3%) (Fig. S2E). Taxa in the *Basidiomycota* were also present across all samples with a mean of 0.47% relative abundance (Fig. S2E, Table 1). At the class level, *Saccharomycetes* was ubiquitous in all samples (Fig. S2F, Table 1), being the dominant class in the mature grape group (87.5%) (Fig. S2F). *Leotiomycetes* (12.9%) and *Dothideomycetes* (5.3%) were also found in the gut contents of both larval groups (Fig. S2F). The classes *Aphelidea*, *Chytridiomycetes*, *Peronosporomycetes*, the clade LKM11 and an incertae sedis class belonging to the *Cryptomycota* were absent in larvae fed on immature grapes but present in those fed on mature grapes (Fig. S2F).

Thirty-two orders were found across all larval samples, with *Saccharomycetales* (83.24%) and *Helotiales* (12.94%) being the most abundant (Fig. S2G, Table 1). The orders *Diaporthales*, *Entylomatales*, *Onygenales*, *Pezizales*, *Russulales* and one unclassified family of the *Leotiomycetes* class were present in larvae fed on mature grapes but absent in larvae fed on immature grapes. In contrast, *Aphelidea*, *Gromochytriales*, *Mortierellales*, *Peronosporomycetes*, *Rhizophydiales*, *Sordariales*, two incertae sedis orders of the *Cryptomycota* phylum and one unclassified order of *Chytridiomycetes* were only present in samples from larvae fed on immature grapes. The dominant order in the GBM-fed mature grape samples was *Saccharomycetales* (87.5%), whereas the clade LKM11 (43.5%) was the most abundant in the larvae fed on immature grapes (Fig. S2G). The subsequent order in terms of abundance was *Helotiales* (12%), which was found in higher quantities in the immature grape-fed larval samples (Fig. S2G).

**Fig. 8.**
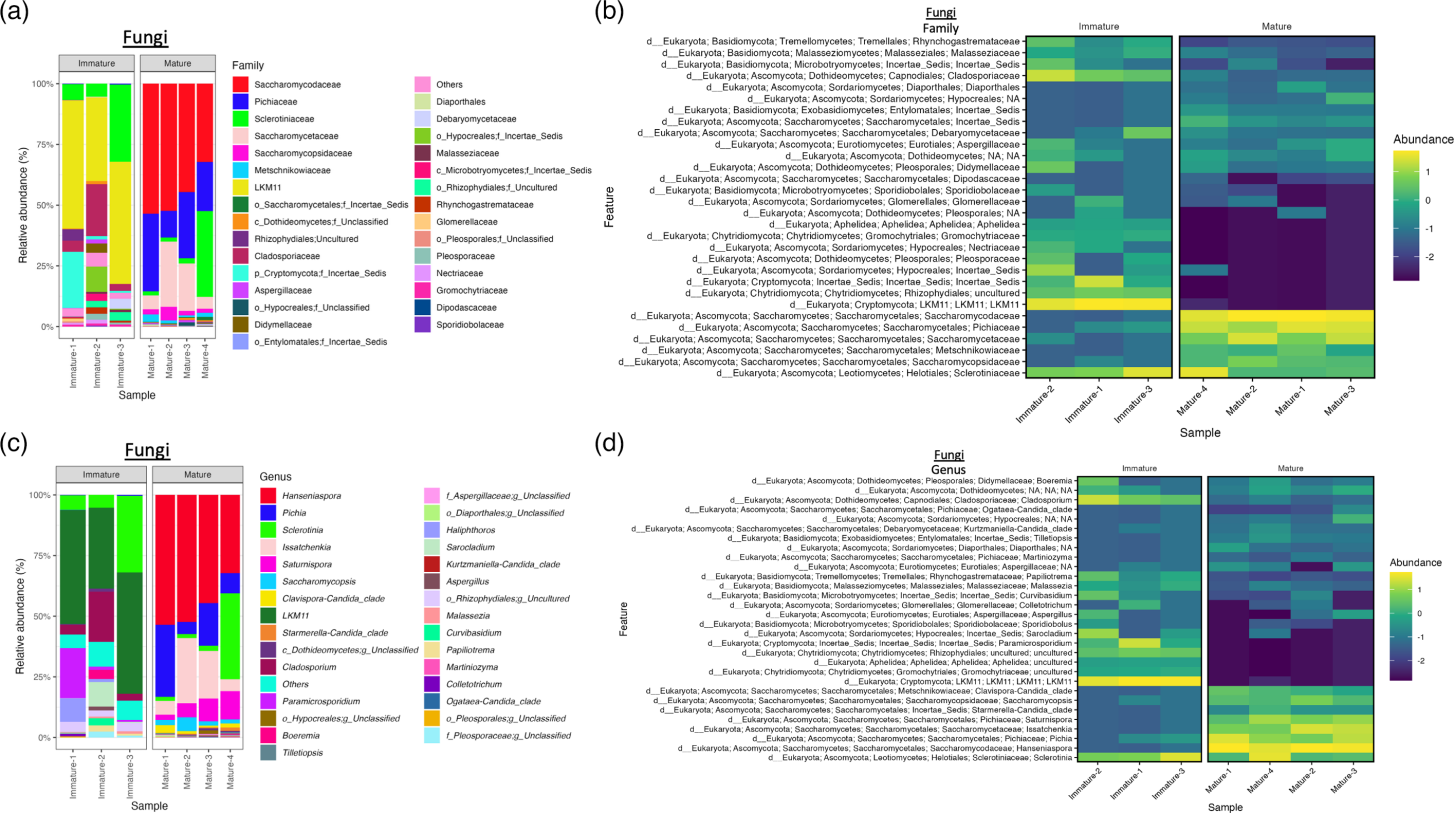
Relative abundance and heat maps of the gut fungal communities of *P. viteana* larvae fed on immature and mature grapes at the (**a, b**) family and (**c, d**) genus levels. (**a, c**) Relative abundance (%) of gut fungal communities across all samples. Taxa below the top 30 abundance cut-off are represented as ‘Others’. ‘Unclassified’ represents taxa that were not assigned to any group. (**b, d**) Heat maps illustrating the 30 most abundant gut fungal communities across samples; colours represent log-transformed relative abundances, where yellow indicates higher and purple indicates lower abundance values.

At the family level (Fig. 8a, b), *Saccharomycodaceae* (45.5%) was the most representative family in the mature grape-fed larval samples, followed by Pichiaceae (22.6%), and to a lesser extent Saccharomycetaceae (14.25%). Conversely, the most abundant taxa at the family level in the immature grape-fed larval samples were the clade LKM11 (43.6%), Sclerotiniaceae (14.2%), Cladosporiaceae (9.2%) and an incertae sedis family belonging to phylum *Cryptomycota* (7.6%). The remaining families had abundances lower than 6% on average. Differential abundance analysis identified 26 families that varied significantly between treatments. Ten families, including *Saccharomycodaceae*, *Metschnikowiaceae* and *Pichiaceae*, were enriched in larvae fed on mature grapes, whereas 16 families, such as *Peronosporomycetes* and *Cladosporiaceae*, were enriched in larvae fed on immature grapes (*q*<0.05, Table S6). From the 43 identified families, 6 were absent in the immature grape-fed larval samples (*Dipodascaceae*, *Metschnikowiaceae*, *Saccharomycodaceae*, two belonged to unclassified families in the orders *Diaporthales* and *Hypocreales*, and two were incertae sedis families from *Entylomatales* and *Saccharomycetales*). In contrast, five families were absent in the mature grape-fed larval samples: *Gromochytriaceae*, *Nectriaceae*, *Pleosporacea*, one uncultured family from *Rhizophydiales* and one incertae sedis from phylum *Cryptomycota*.

At the genus level, the classification rate was 79.5%. Eight genera were absent in the immature grape-fed larval samples: *Clavispora*–*Candida* clade, *Hanseniaspora*, *Issatchenkia*, *Martiniozyma*, *Ogataea*–*Candida_clade*, *Starmerella*–*Candida_clade*, *Tilletiopsis* and one unclassified genus from the order *Hypocreales*. Four genera were absent in the mature grape-fed larval samples: *Haliphthoros*, *Paramicrosporidium* and two unclassified genera from the *Pleosporacea* family and the *Rhizophydiales* order. Differential abundance analysis identified 27 genera that varied significantly between treatments. Twenty fungi genera, including *Hanseniaspora*, *Saturnispora* and *Issatchenkia,* were enriched in larvae fed on mature grapes, whereas seven genera, such as LKM11, were enriched in larvae fed on immature grapes (*q*<0.05, Table S7). The most abundant genus in the mature and immature grape-fed larval samples was *Hanseniaspora* (45.7%) and the clade LKM11 (43.5%), respectively (Fig. 8c, d). The top four genera with the highest abundance in the mature grape-fed samples were *Hanseniaspora, Pichia, Sclerotinia* and *Issatchenkia* (Fig. 8d).

**Fig. 9.**
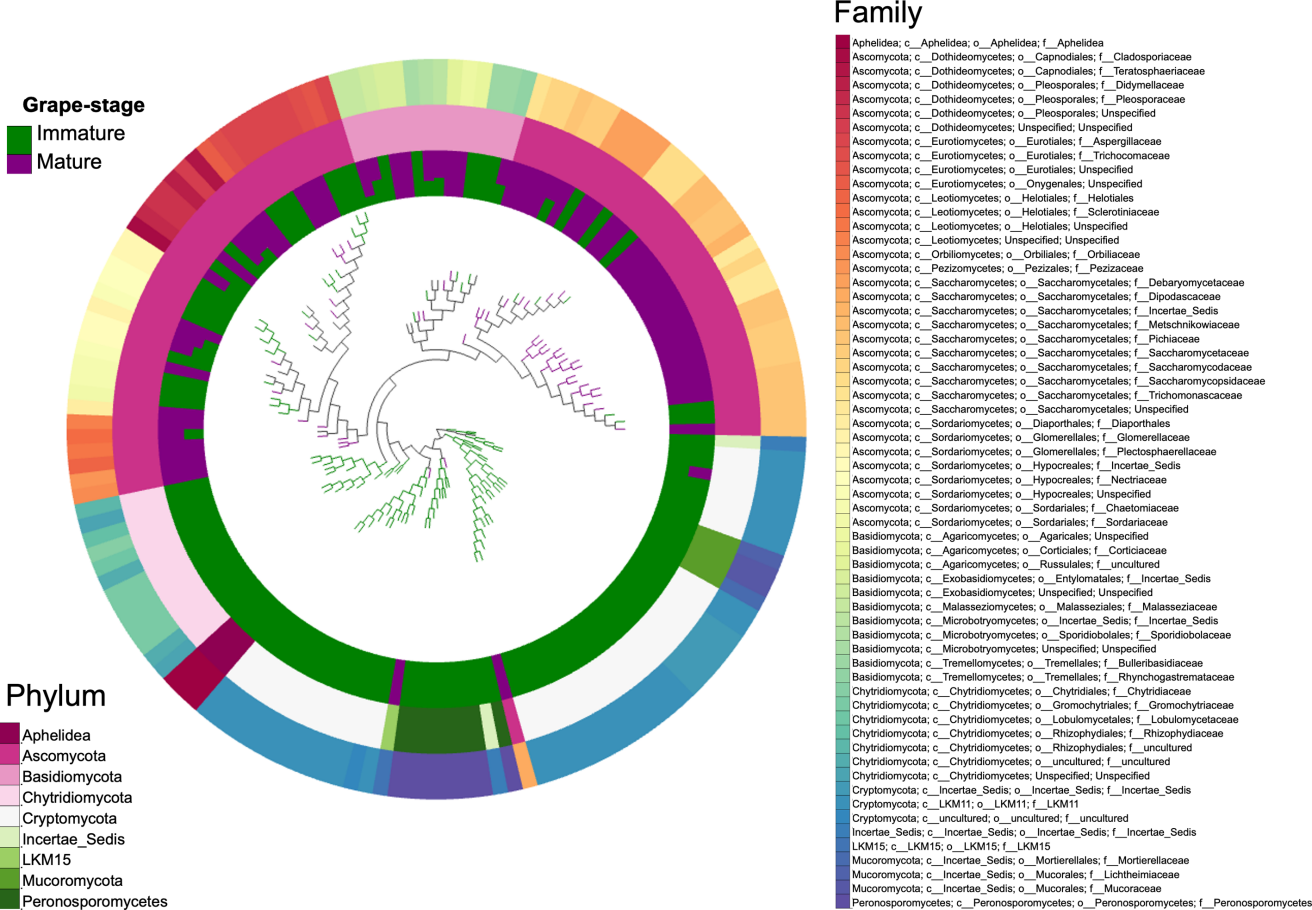
Phylogenetic tree of fungal ASVs generated from 18S rRNA gene sequences of *P. viteana* larval guts fed on immature and mature Concord grapes. The phylogenetic tree represents features at the ASV feature level. The phylogenetic tree depicts relationships among ASVs (features), and branch colours indicate the treatment group (guts from larvae fed mature or immature grapes) in which each ASV was detected. The inner concentric ring indicates the grape stage. The middle ring represents taxonomic assignment at the phylum level, with colours corresponding to the bottom left legend. The outer ring shows taxonomic assignment at the family level, following the colour palette displayed on the right.

The microbiota of GBM larval guts, based on the 18S rRNA analysis, was divided into four clades (Fig. 9). Two clades were formed by the *Ascomycota*, which was the most abundant phylum across all larval samples. The first clade comprised the order *Saccharomycetales*, containing a subclade of four families (*Metschnikowiaceae*, *Pichiaceae*, *Saccharomycetaceae* and *Incertae sedis*) mostly detected in the guts of individuals fed on mature grapes. The second *Ascomycota* clade was divided into two subclades. The first one comprised the orders *Capnodiales*, *Pleosporales* and *Eurotiales*, found in both larval treatment groups. The second sub-clade contained the orders *Diaporthales*, *Glomerellales*, *Hypocreales*, *Sordariales*, *Helotiales*, *Orbiliales* and the *Pezizales*, most of which were found in both larval treatment groups. The second most abundant phylum, *Basidiomycota*, comprised the third clade, and its features were detected in both larval treatment groups. The fourth and biggest clade in the fungal phylogenetic tree was formed by the phyla *Cryptomycota*, *Chytridiomycota*, *Mucoromycota*, *Peronosporomycetes* and LKM15. All of them were exclusively found in larvae fed on immature grapes, whereas the phylum LKM15 was only found in samples from the mature grape group.

## Discussion

The gut microbiome of GBM larvae fed on Concord grapes contains a diverse array of bacterial and fungal symbionts. The families *Rickettsiaceae* and *Enterobacteriaceae* within the phylum *Proteobacteria* contained the most abundant bacterial taxa. The most abundant fungi were in the LKM11 clade within the phylum *Cryptomycota*, which are not typically associated with insect guts and likely reflect environmental taxa ingested with grape tissue rather than true gut residents. The most abundant yeasts were in the order *Saccharomycetales*. We did not find significant differences in the diversity of GBM gut-associated bacteria between larvae fed on mature and immature grapes; however, differential abundance analysis revealed significant shifts in specific bacterial taxa between treatments. In contrast, fungal communities showed broader differences across multiple taxonomic levels. These results support our hypothesis of higher microbial abundance in larvae fed on mature grapes. To our knowledge, this is the first microbiota survey from *P. viteana* guts and the first on fruit-borer insects fed on fruit at different ripening stages.

### GBM gut-associated bacteria

*Proteobacteria* were the most abundant bacterial phylum in *P. viteana* larval guts, followed by *Cyanobacteria*, *Firmicutes*, *Bacteroidetes* and *Actinobacteria* (Table 1). These phyla are among the most abundant endosymbionts found in lepidopteran larval guts [3,46,50]. *Proteobacteria* are likewise reported as part of the core microbiome in some lepidopteran fruit-borers. In a four-species comparison of the oriental fruit moth (*G. molesta*), the peach fruit borer (*Conogethes punctiferalis*), the peach fruit moth (*Carposina sasakii*) and the codling moth (*Cydia pomonella*), *Proteobacteria* and *Firmicutes* were the dominant phyla in all the species, whereas *Actinobacteria* and *Bacteroidetes* were present in lower abundance [26]. A more detailed survey of the tortricid *G. molesta* confirmed this pattern across its entire life cycle: *Proteobacteria* and *Firmicutes* were the dominant phyla in the gut of every ontogenetic stage, with genera such as *Gluconobacter*, *Pantoea*, *Enterococcus* and *Enterobacter* successively prevailing as the larvae matured [51]. These convergent findings indicate that fruit-feeding tortricids host gut microbiota enriched in *Proteobacteria*–*Firmicutes* lineages, mirroring the phylum-level profile observed in our GBM–Concord–grape system.

*Wolbachia* (Family Rickettsiaceae), the most abundant genus identified in this study, are primarily reproductive parasites commonly found in insects [52]. *Wolbachia* infections are common in Lepidoptera, manipulating reproduction and, in one species, increasing susceptibility to baculovirus infections [53,55]. The tortricids *Homona magnanima* and *Lobesia botrana* also harbour *Wolbachia* symbionts [56,57]. In *H. magnanima*, closely related *Wolbachia* strains have been associated with distinct reproductive phenotypes, including male-killing and non-male-killing, highlighting the important role of symbiont genetic variation in shaping host reproductive manipulation [58]. This highlights the need to further characterize the *Wolbachia* strains in *P. viteana* populations, although sex ratio distortions were not identified in recent studies [34]. Beyond its well-known reproductive effects, *Wolbachia* can also influence host-associated microbial communities by altering bacterial diversity, relative abundance and community composition in arthropod hosts [59]. Although *Wolbachia* has been detected in gut samples of several insect orders, including Lepidoptera [60], its localization and functional role in the gut remain poorly understood in this group. In the parasitoid *Spalangia cameroni* (Hymenoptera: Spalangiidae), *Wolbachia* were found colonizing gut epithelia, malpighian tubules and other structures [61], but their functional role in these structures remains unclear. Studies in *D. melanogaster* further demonstrated that *Wolbachia* can affect the abundance of *Acetobacter* and *Lactobacillus* in the gut but had no effect on host immunity [62]. These results highlight the need for further research to elucidate the role of *Wolbachia* infections in the composition and function of insect gut microbiomes. Detailed functional studies will be needed to determine the specific role of *Wolbachia* in GBM.

The alpha and beta diversity analyses indicated that the composition of gut bacteria is similar between GBM larvae feeding on immature and mature grapes, and the exclusion of *Wolbachia* sequences did not change this outcome (Table S4). These results were unexpected due to the well-documented close association of bacterial composition with diet type in lepidopterans [3,63, 64] and the prominent biochemical differences between ripe and unripe grapes [35]. As measured in this study, ripe Concord grapes had 3.2 times more sugars and were 1.2 times less acidic (pH) than unripe grapes. However, the acidity of the grape pulp remained very high through berry development for most bacteria to grow (pH<3.19). Perhaps the alkaline pH common in the guts of Lepidoptera larvae (but unknown in GBM) counteracts the acidity of the ingested food. Differential abundance analysis identified several bacterial families that varied between larvae fed on immature and mature grapes (*q*<0.05, Table S2). Among these, two families enriched in mature-grape-fed larvae stood out for their ecological relevance: *Acetobacteraceae* and an unclassified lineage within the order *Bacteroidales*. *Acetobacteraceae*, which includes the well-characterized acetic acid bacteria, is well adapted to sugar-rich, low-pH environments such as those found in ripe fruits and fermented products [65,66], and its higher abundance in larvae fed on mature Concord grapes is consistent with the marked increase in sugar content observed after veraison. Members of *Bacteroidales* have been repeatedly detected across a broad range of insect microbiomes [23,67], where they are thought to contribute to polysaccharide degradation, fermentation and nutrient acquisition. At the genus level, both *Gluconacetobacter* and *Gluconobacter* – the two most abundant genera within *Acetobacteraceae* detected in this study – were significantly enriched in larvae fed on mature grapes (*q*<0.05). *Gluconacetobacter* has been reported in the gut microbiome of the spotted-wing drosophila, *Drosophila suzukii* [68], a frugivorous fly that, like GBM, develops in ripe or overripe fruit. *Gluconobacter* has similarly been found across a range of fruit-feeding insects, including *G. molesta*, *Carposina sasakii*, *D. suzukii* and *D. melanogaster* [69,71], and was shown to be more abundant in the carposphere of ripe compared to unripe *Vitis vinifera* grapes cv. Riesling in a previous microbiome survey [72]. Together, these patterns suggest that the enrichment of *Acetobacteraceae* genera in GBM larvae may be diet-mediated, with larvae acquiring these bacteria from the microbial community present in ripe grape tissue. Members of *Acetobacteraceae* and the order *Bacteroidales* have been commonly detected in the guts of diverse insect species, where they may establish functionally important associations with hosts, although environmental acquisition likely contributes substantially to their transmission.

The functional profile of the GBM larval gut bacterial community predicted by PICRUSt2 identified enriched pathways involved in carbohydrate breakdown, amino acid biosynthesis and detoxification of plant-derived compounds (Fig. 6). The two most abundant pathways: tricarboxylic acid (TCA) cycle IV (2-oxoglutarate decarboxylase) and TCA cycle V (2-oxoglutarate: ferredoxin oxidoreductase) are part of the TCA routes characteristic of acetic-acid bacteria (*Acetobacteraceae*) such as *Gluconobacter* and *Acetobacter* [66,73, 74], which prevailed in larvae fed on ripe grapes. A similar acetate-driven TCA shunt has been documented in the *Acetobacteraceae Snodgrassella alvi* inhabiting honeybee guts and other sugar-rich niches, where rapid but incomplete carbohydrate oxidation is favoured [75]. The super pathway of pyrimidine deoxyribonucleotide *de novo* route is widely conserved across all domains of life and serves as a fundamental housekeeping route, supplying the essential uridine monophosphate and deoxythymidine monophosphate precursors required for DNA replication and repair [76]. PICRUSt surveys of *G. molesta* predicted amino-acid metabolism and DNA replication/repair as major functional categories [50,51], suggesting a conserved nutritional role for enterobacterial symbionts in frugivorous Lepidoptera. Similarly, predictive functional profiling in other lepidopterans has highlighted pathways related to carbohydrate, amino acid, lipid and xenobiotic metabolism, supporting a potential role for gut bacteria in nutrition and detoxification processes in this insect order [77]. Despite these insights, the functional characterization presented here should be viewed as hypotheses. PICRUSt2 infers pathways from 16S rRNA sequences by matching each ASV to the closest reference genome; however, this approach has several limitations, including overestimated accuracy (simple correlation metrics remain high even on permuted data), optimal performance only in well‐characterized microbiomes (human), failure to detect many genes detected by true metagenomes and sharply reduced inference accuracy outside human samples. Therefore, individual pathway predictions, especially in underrepresented environments or for non-housekeeping functions, should be interpreted with caution [78]. Additionally, many *P. viteana* symbionts found in this study lack close cultured relatives; therefore, attributed functions may be unreliable. Future work employing shotgun metagenomics and metatranscriptomics could improve bacterial functional predictions.

### GBM gut-associated fungi

Fungi are crucial players in insect–plant interactions, although they have often been overlooked [1]. Gut-associated yeast communities have been reported in Diptera [79,81], Hymenoptera [82,83], Coleoptera [84,86], Blattodea (suborder Isoptera) [87,88], Hemiptera [89,91], Neuroptera [92,93] and to a lesser extent in some Lepidoptera [4,94, 95]. Most yeasts found in insects belong to the phylum *Ascomycota* [96]. Ascomycota yeasts are part of a wide variety of microorganisms that have been reported in multiple microbial grape carposphere-associated surveys worldwide [97,99]. Changes in grape composition throughout the ripening process, such as the increase in Brix degrees, provide sugar-rich environments that are very favourable for the growth and development of these fungi within ripe grapes. Our results showed that the majority of the classified features found in the guts of GBM larvae fed on mature grapes belonged to the budding yeasts (phylum *Ascomycota*, class *Saccharomycetes*) with a strong enrichment of the families *Saccharomycodaceae*, *Saccharomycetaceae*, *Pichiaceae*, *Metschnikowiaceae* and *Saccharomycopsidaceae* (*q*<0.05). Multiple genera within this group are commonly associated with sugar-rich environments like fruits and fermented products. In particular, GBM guts were enriched with *Hanseniaspora*, *Pichia*, *Issatchenkia*, *Saturnispora* and *Saccharomycopsis* genera *that* have been isolated from grape berries and grape must, where they contribute to fermentation and sugar metabolism [100,103]. Hanseniaspora are known to mediate the early stages of spontaneous fruit fermentation and add desirable volatile compounds to wine aroma, but they can also affect the fermentation quality by producing acetic acid [104]. Pichia are considered a low- or non-fermentative yeast and are used in enology to increase the quality of specific varietal wines [105]. Notably, several yeast genera identified in GBM guts, including *Issatchenkia*, *Saccharomycopsis*, *Pichia* and *Zygoascus*, have been associated with the sour rot disease complex in grapevines [106], consistent with the late-season collection of our samples, when berries may already exhibit early signs of decay. Therefore, most GBM-gut-associated yeasts might be either environmental or pathogenic opportunistic micro-organisms of rotten grapes.

Some *Saccharomycetes* have interactions with bacteria that may be beneficial for fruit-feeding insects but may also contribute to the development of fruit diseases. Previous studies have demonstrated that some yeast strains improve *D. suzukii* larval development and survival, likely by nutrient provisioning [107], as it has been demonstrated in *D. melanogaster* [108]. However, when feeding on overripe fruits, the fermentation mediated by yeasts produces alcohol, which is detrimental to living tissues. This may be alleviated in insects by acetic acid bacteria, like *Gluconobacter* and *Acetobacter,* that metabolize alcohol into acetic acid. Fischer and collaborators [109] demonstrated this mechanism in *D. melanogaster.* Furthermore, these insects prefer a mixture of *Saccharomyces* and *Acetobacter* to the same micro-organisms grown separately. Similarly, the pathogenic fungus *Botrytis cinerea* has beneficial effects on the development and oviposition behaviour of the European grapevine moth, *L. botrana*. Females prefer to lay their eggs on grape-infected berries and exhibit higher fitness than on uninfected grapes [110]. When grapes are consumed, the fungus is also ingested, and this serves as a substantial source of precursor sterols for the larvae to use in the synthesis of growth- and development-promoting hormones [111]. Although in our study we found higher levels of Acetobacteraceae bacteria and *Saccharomycetales* (*Saccharomycetaceae*, *Saccharomycodaceae*) yeasts in larvae fed on mature grapes, the existence of an interaction between these micro-organisms and their effects on GBM larvae and sour rot development has not been investigated.

The most abundant fungi groups in GBM larval guts fed on immature grapes were *Cryptomycota* and *Ascomycota* (Table 1). This pattern may reflect differences in the microbial communities associated with grapes at different ripening stages, as immature and mature berries are known to harbour distinct fungal assemblages, with ripening favouring yeast-dominated communities. *Cryptomycota* is a phylum of mostly undescribed and deeply diverging fungi found in diverse environments [112]. Carmichael *et al*. [113] reported *Cryptomycota* communities associated with the grey mould *B. cinerea* in some vineyards in South Africa. The fungi clade LKM11, which was the most abundant member of the *Cryptomycota* phylum identified in this study, has been found in various environmental (terrestrial and marine) DNA surveys worldwide [114]. Importantly, *Cryptomycota* are not typically associated with insect gut microbiomes, and their presence here most likely reflects environmental taxa ingested with grape tissue rather than true gut residents. Because these fungi are widespread saprobes, LKM11 cells may simply traverse the gut as resistant spores or vegetative fragments incidentally ingested with grape tissue, rather than acting as persistent, active symbionts. In addition, the limited taxonomic resolution and potential bias of the 18S V4 marker may have contributed to the apparent enrichment of this group in immature samples.

Beyond the LKM11 clade, the most abundant families in immature-fed larvae were *Sclerotiniaceae* and *Cladosporiaceae*. *Sclerotinia* spp., the dominant genus within *Sclerotiniaceae*, are necrotrophic soil-borne pathogens capable of infecting grapevine tissues and causing shoot blight or rot under cool and humid conditions [115]. *Cladosporiaceae*, which includes both foliar pathogens and common grapevine endophytes [116], was also significantly enriched in immature-fed larvae (*q*<0.05). The presence of both groups in larval guts most likely reflects passive ingestion of fungal material from infected or colonized immature berry tissue, as unripe grapes are known to harbour distinct fungal pathogen assemblages compared to ripe berries [72]. Taken together, these patterns suggest that the GBM gut mycobiome is largely transient and diet-derived, mirroring the fungal communities present in grape tissue at each ripening stage rather than representing a stable, host-associated community [18].

### Study limitations

Larvae were collected directly from vineyard grapes; therefore, surface and pulp microbes could not be sterilized without disturbing the natural diet. In addition, the number of biological replicates was limited (four immature and three mature samples, each sample comprising a pool of 10 GBM larval guts), which may reduce statistical power and limit the detection of subtle differences in microbial community composition. Although pooling helped obtain sufficient DNA and may reduce individual-level variability, it does not fully compensate for the low number of biological replicates. Reagent blanks, surface-sterilization checks and grape-pulp controls were, therefore, not included, meaning some diet-derived taxa appear in gut profiles. Moreover, all samples were taken from a single vineyard in one growing season; geographic or inter-annual variation in environmental microbiota could not be assessed, and our results may not translate to other GBM populations. Community profiles were generated with short 16S V4 and 18S amplicons (341F/806R and 528F/706R primers), which are subject to primer bias and provide limited taxonomic resolution at the species level. The 16S V3–V4 region offers moderate resolution for bacteria, whereas the 18S V4 region has limited discriminatory power for fungi and may underrepresent certain groups (e.g. non-*Ascomycota*) or co-amplify non-fungal eukaryotic DNA. Therefore, taxonomic assignments below the genus level were not included. Predicted pathways were inferred with PICRUSt2 rather than measured directly; therefore, functional assignments remain hypothetical until validated by metagenomics, metatranscriptomics or metabolomics.

## Conclusions

Our study reveals the structure of the gut bacterial and fungal communities of GBM larvae fed on grapes at two different ripening stages. *Wolbachia* was the dominant bacterial genus found in our samples. While bacterial community composition and diversity did not differ significantly between treatments, differential abundance analysis revealed specific bacterial taxa enriched in each larval group. In contrast, fungal communities showed markedly broader differences: the total abundance of fungal taxa in larvae fed on mature grapes was 10-fold higher than in those fed on immature grapes, with significant compositional shifts detected across multiple taxonomic levels. Mature-grape larvae were enriched in *Saccharomycetales* yeasts, whereas immature-grape larvae were dominated by *Cryptomycota* (LKM11), and these differences could be linked to the grape cluster’s own microbial communities. Some gut-associated bacteria may have potential roles in the GBM larvae’s metabolic functions, such as carbohydrate metabolism, amino-acid biosynthesis and detoxification of plant phenolics, as implied by PICRUSt2 predictions, but these functions remain to be experimentally validated. Additionally, further research should consider assessing the influence of the environment and grape cultivar on the GBM gut microbiome composition, including multi-vineyard and multi-year sampling. Integrating functional ‘omics and manipulative experiments could help determine promising microbial taxa or pathways to target for sustainable GBM management.

## Supplementary material

10.1099/mgen.0.001756 Supplementary Material 1.

## Abbreviations

ANCOM-BC: Analysis of Composition of Microbiomes with Bias Correction
ASV: amplicon sequence variant
FDR: false discovery rate
GBM: grape berry moth
KEGG: Kyoto Encyclopedia of Genes and Genomes
NCBI: National Center for Biotechnology Information
NMDS: non-metric multidimensional scaling
PCoA: principal coordinate analysis
PERMANOVA: permutational multivariate ANOVA
PICRUSt: Phylogenetic Investigation of Communities by Reconstruction of Unobserved States
QIIME: Quantitative Insights Into Microbial Ecology
RA: relative abundance
TCA: tricarboxylic acid
