# Supplementary material for: Gut microbiome of the grape berry moth, Paralobesia viteana (Lepidoptera: Tortricidae) larvae through the grape ripening process revealed by high-throughput 16S and 18S rRNA sequencing
Source: Microb Genom. 2026 Jun 17;12(6):001756. doi: 10.1099/mgen.0.001756 (PMC13274764; doi:10.1099/mgen.0.001756)
Supplement: Supplementary Material 1. [file mgen-12-01756-s001.pdf]

Supplementary Information

Article title: Gut microbiome of the grape berry moth, *Paralobesia viteana* (Lepidoptera: Tortricidae) larvae through the grape ripening process revealed by high-throughput 16S and 18S rRNA sequencing

Authors:
Laura Laiton*
Pennsylvania State University, Department of Entomology, University Park, PA, 16802, United States
lal5792@psu.edu
ORCID Number: 0000-0002-3536-0386

Flor E. Acevedo*
Pennsylvania State University, Department of Entomology, University Park, PA, 16802, United States
fea5007@psu.edu
ORCID Number: 0000-0002-0946-9951

*Corresponding authors.

Supplementary tables:

Table S1. Quality control Statistics of the 16S rRNA gene sequences of GBM gut microbiota from larvae fed on immature and mature Concord grapes.

Sample-id	Input	Filtered	Percent of input passed filter	Denoised	Merged	Percent of input merged	Non-chimeric	Percent of input non-chimeric
Immature-1	172,456	145,826	84.56	142,906	135,379	78.5	113,520	65.83
Immature-2	183,159	153,333	83.72	151,381	145,395	79.38	125,030	68.26
Immature-3	178,055	151,909	85.32	149,906	144,574	81.2	118,531	66.57
Immature-4	171,669	147,549	85.95	145,200	139,836	81.46	109,435	63.75
Mature-1	187,314	158,625	84.68	156,844	152,125	81.21	133,690	71.37
Mature-2	187,929	162,275	86.35	159,853	152,766	81.29	135,144	71.91
Mature-3	185,581	159,501	85.95	155,545	145,115	78.19	127,785	68.86

Table S2. Differential abundance analysis of bacterial taxa at the family level from GBM larvae fed on grapes at two developmental stages (Immature vs. Mature) using ANCOM-BC (Analysis of Compositions of Microbiomes with Bias Correction) implemented in QIIME 2. Only taxa with a p-value < 0.05 are shown.

Family	LFC	SE	W	p value	q value	Enriched in	Significant
Leptotrichiaceae	2.8673	0.3941	7.2756	3.45E-13	8.62E-11	Mature	Yes
o__Bacteroidales;f__Unclassified	1.9843	0.3266	6.0750	1.24E-09	3.09E-07	Mature	Yes
Rhizobiaceae	-1.1595	0.2109	-5.4974	3.85E-08	9.56E-06	Immature	Yes
Acetobacteraceae	2.4291	0.5175	4.6939	2.68E-06	6.62E-04	Mature	Yes
[Paraprevotellaceae]	2.4915	0.6146	4.0538	5.04E-05	0.0124	Mature	Yes
Sphingomonadaceae	1.6012	0.4658	3.4378	5.86E-04	0.1437	Mature	No
Enterobacteriaceae	-0.9320	0.2731	-3.4123	6.44E-04	0.1572	Immature	No
c__Alphaproteobacteria;o__Unclassified;f__Unclassified	2.9260	0.9048	3.2338	0.0012	0.2969	Mature	No
Neisseriaceae	2.5991	0.8066	3.2222	0.0013	0.3079	Mature	No
Christensenellaceae	-1.2250	0.3817	-3.2094	0.0013	0.3206	Immature	No
o__0319-7L14;f__Unclassified	1.6484	0.5586	2.9513	0.0032	0.7595	Mature	No
Caulobacteraceae	2.0740	0.7372	2.8132	0.0049	1	Mature	No
Gaiellaceae	1.7673	0.6316	2.7981	0.0051	1	Mature	No
[Weeksellaceae]	-1.5372	0.5572	-2.7589	0.0058	1	Immature	No
Coxiellaceae	1.9659	0.7172	2.7413	0.0061	1	Mature	No
Planococcaceae	1.7386	0.6439	2.7002	0.0069	1	Mature	No
o__N1423WL;f__Unclassified	0.8647	0.3230	2.6766	0.0074	1	Mature	No
o__Actinomycetales;f__Unclassified	0.8647	0.3230	2.6766	0.0074	1	Mature	No
Rubrobacteraceae	0.8647	0.3230	2.6766	0.0074	1	Mature	No
HTCC2089	0.8647	0.3230	2.6766	0.0074	1	Mature	No
Nocardiopsaceae	0.8647	0.3230	2.6766	0.0074	1	Mature	No
Alicyclobacillaceae	0.8647	0.3230	2.6766	0.0074	1	Mature	No
Corynebacteriaceae	1.4252	0.5371	2.6532	0.0080	1	Mature	No
S24-7	1.7053	0.6457	2.6409	0.0083	1	Mature	No
o__Bacillales;f__Unclassified	0.9390	0.3661	2.5647	0.0103	1	Mature	No
c__SAR202;o__Unclassified;f__Unclassified	0.9390	0.3661	2.5647	0.0103	1	Mature	No
o__[Pedosphaerales];f__Unclassified	0.9390	0.3661	2.5647	0.0103	1	Mature	No
o__Chloroflexales;f__Unclassified	0.9390	0.3661	2.5647	0.0103	1	Mature	No
Syntrophobacteraceae	0.9390	0.3661	2.5647	0.0103	1	Mature	No
o__Solirubrobacterales;f__Unclassified	0.9390	0.3661	2.5647	0.0103	1	Mature	No
OM27	0.9998	0.4047	2.4704	0.0135	1	Mature	No
HTCC2188	0.9998	0.4047	2.4704	0.0135	1	Mature	No
Xanthomonadaceae	-0.6503	0.2655	-2.4493	0.0143	1	Immature	No
Piscirickettsiaceae	2.2748	0.9431	2.4121	0.0159	1	Mature	No

Hyphomicrobiaceae	1.9859	0.8253	2.4064	0.0161	1	Mature	No
o__MND1;f__Unclassified	1.0512	0.4391	2.3941	0.0167	1	Mature	No
c__ML635J-21;o__Unclassified;f__Unclassified	1.0512	0.4391	2.3941	0.0167	1	Mature	No
Conexibacteraceae	1.0512	0.4391	2.3941	0.0167	1	Mature	No
Xenococcaceae	1.0512	0.4391	2.3941	0.0167	1	Mature	No
Lactobacillaceae	1.3591	0.5743	2.3665	0.0180	1	Mature	No
o__Clostridiales;f__Unclassified	1.3135	0.5568	2.3591	0.0183	1	Mature	No
Kineosporiaceae	1.0957	0.4698	2.3322	0.0197	1	Mature	No
c__Gemm-2;o__Unclassified;f__Unclassified	1.0957	0.4698	2.3322	0.0197	1	Mature	No
Erythrobacteraceae	2.0136	0.8726	2.3075	0.0210	1	Mature	No
Campylobacteraceae	1.8532	0.8097	2.2887	0.0221	1	Mature	No
Trueperaceae	1.1350	0.4976	2.2810	0.0226	1	Mature	No
Actinomycetaceae	1.5540	0.6855	2.2670	0.0234	1	Mature	No
Euzebyaceae	1.4927	0.6635	2.2497	0.0245	1	Mature	No
c__Gitt-GS-136;o__Unclassified;f__Unclassified	1.1701	0.5228	2.2380	0.0252	1	Mature	No
Vibrionaceae	1.1350	0.5217	2.1757	0.0296	1	Mature	No
o__Bacillales;f__Unclassified	1.2575	0.5871	2.1421	0.0322	1	Mature	No
Rikenellaceae	1.3444	0.6347	2.1181	0.0342	1	Mature	No
Actinosynnemataceae	1.2822	0.6055	2.1177	0.0342	1	Mature	No
Rhodobiaceae	1.2822	0.6055	2.1177	0.0342	1	Mature	No
Porphyromonadaceae	0.6245	0.2970	2.1026	0.0355	1	Mature	No
c__Gammaproteobacteria;o__Unclassified;f__Unclassified	1.3052	0.6228	2.0959	0.0361	1	Mature	No
Desulfobulbaceae	1.7322	0.8282	2.0916	0.0365	1	Mature	No
c__Betaproteobacteria;o__Unclassified;f__Unclassified	1.3055	0.6303	2.0714	0.0383	1	Mature	No
Rhodothermaceae	1.3470	0.6543	2.0587	0.0395	1	Mature	No
Geodermatophilaceae	1.4174	0.7080	2.0019	0.0453	1	Mature	No
[Chthoniobacteraceae]	1.9899	0.9980	1.9939	0.0462	1	Mature	No
Ruminococcaceae	0.7615	0.3843	1.9813	0.0476	1	Mature	No
o__Rhodospirillales;f__Unclassified	1.4619	0.7423	1.9694	0.0489	1	Mature	No
o__AKYG1722;f__Unclassified	1.4619	0.7423	1.9694	0.0489	1	Mature	No
Flammeovirgaceae	1.4755	0.7528	1.9600	0.0500	1	Mature	No

LFC, log fold-change estimated by ANCOM-BC; positive values indicate enrichment in the Mature stage relative to Immature. SE, standard error of the LFC estimate. W, Wald test statistic (LFC/SE). p-value, nominal p-value from the Wald test. q-value, p-value adjusted for multiple testing using the Benjamini–Hochberg false discovery rate (FDR) procedure. Enriched in, the grape developmental stage in which the taxon shows higher relative abundance based on the direction of the LFC. Significant, whether the taxon passed FDR correction at $q < 0.05$ (Yes) or was nominally significant but did not survive multiple testing correction (No). Unclassified or partially annotated taxa are shown with their lowest available taxonomic assignment. Brackets around a family name indicate provisional taxonomic designations in the original Greengenes taxonomy.

Table S3. Differential abundance analysis of bacterial taxa at the genus level from GBM larvae fed on grapes at two developmental stages (Immature vs. Mature) using ANCOM-BC (Analysis of Compositions of Microbiomes with Bias Correction) implemented in QIIME 2. Only taxa with a p-value < 0.05 are shown.

Genus	LFC	SE	W	p value	q value	Enriched in	Significant
Leptotrichia	2.6139	0.2829	9.2389	2.49E-20	1.10E-17	Mature	Yes
Gluconacetobacter	3.4305	0.3873	8.8563	8.27E-19	3.63E-16	Mature	Yes
o__Bacteroidales;f__;g__Unclassified	1.9478	0.3115	6.2526	4.04E-10	1.77E-07	Mature	Yes
Agrobacterium	-1.2286	0.2419	-5.0780	3.81E-07	1.67E-04	Immature	Yes
Neisseria	2.1381	0.4435	4.8211	1.43E-06	6.23E-04	Mature	Yes
Gluconobacter	2.3626	0.4904	4.8179	1.45E-06	6.31E-04	Mature	Yes
f__Christensenellaceae;g__Unclassified	-1.2615	0.3130	-4.0309	5.56E-05	0.0241	Immature	Yes
[Prevotella]	2.6096	0.6513	4.0071	6.15E-05	0.0266	Mature	Yes
Peptostreptococcus	1.2687	0.3290	3.8565	1.15E-04	0.0497	Mature	Yes
CF231	2.6932	0.6984	3.8563	1.15E-04	0.0497	Mature	Yes
Stenotrophomonas	-1.4117	0.3773	-3.7412	1.83E-04	0.0787	Immature	No
Erwinia	-1.5167	0.4068	-3.7287	1.92E-04	0.0826	Immature	No
Frateuria	2.5233	0.7020	3.5945	3.25E-04	0.1391	Mature	No
Filifactor	1.6633	0.4778	3.4810	5.00E-04	0.2133	Mature	No
Acetobacter	2.2140	0.6491	3.4110	6.47E-04	0.2757	Mature	No
f__Rikenellaceae;g__Unclassified	1.3821	0.4324	3.1966	0.001	0.5910	Mature	No

c__Alphaproteobacteria;o__Unclassified;f__Unclassified;g__Unclassified	2.8895	0.9112	3.1711	0.002	0.6438	Mature	No
Hymenobacter	2.1321	0.6800	3.1353	0.002	0.7263	Mature	No
Selenomonas	1.6564	0.5341	3.1011	0.002	0.8135	Mature	No
Coxiella	2.2761	0.7340	3.1009	0.002	0.8135	Mature	No
o__0319-7L14;f__;g__Unclassified	1.6119	0.5211	3.0936	0.002	0.8306	Mature	No
f__Clostridiaceae;g__Unclassified	1.9028	0.6244	3.0473	0.002	0.9674	Mature	No
f__Ruminococcaceae;g__Unclassified	1.2126	0.4065	2.9833	0.003	1	Mature	No
Sphingomonas	1.5163	0.5099	2.9737	0.003	1	Mature	No
Chryseobacterium	-1.5737	0.5399	-2.9148	0.004	1	Immature	No
f__S24-7;g__Unclassified	1.6688	0.5772	2.8911	0.004	1	Mature	No
Escherichia	2.3531	0.8175	2.8783	0.004	1	Mature	No
Tatumella	2.7637	0.9804	2.8190	0.005	1	Mature	No
Devosia	2.0017	0.7130	2.8073	0.005	1	Mature	No
f__Gaiellaceae;g__Unclassified	1.7308	0.6323	2.7374	0.006	1	Mature	No
Ruminococcus	1.1044	0.4203	2.6277	0.009	1	Mature	No
Paludibacter	1.5956	0.6083	2.6230	0.009	1	Mature	No
f__Paenibacillaceae;g__Unclassified	0.8281	0.3164	2.6169	0.009	1	Mature	No
f__Planococcaceae;g__Unclassified	0.8281	0.3164	2.6169	0.009	1	Mature	No
o__N1423WL;f__Unclassified;g__Unclassified	0.8281	0.3164	2.6169	0.009	1	Mature	No
o__Actinomycetales;f__Unclassified;g__Unclassified	0.8281	0.3164	2.6169	0.009	1	Mature	No
Rubrobacter	0.8281	0.3164	2.6169	0.009	1	Mature	No
f__HTCC2089;g__Unclassified	0.8281	0.3164	2.6169	0.009	1	Mature	No
Streptomonospora	0.8281	0.3164	2.6169	0.009	1	Mature	No
Alicyclobacillus	0.8281	0.3164	2.6169	0.009	1	Mature	No
Capnocytophaga	1.3035	0.4991	2.6115	0.009	1	Mature	No
Coprococcus	1.6896	0.6491	2.6028	0.009	1	Mature	No
Corynebacterium	1.3886	0.5431	2.5567	0.011	1	Mature	No
f__Veillonellaceae;g__Unclassified	1.0592	0.4195	2.5245	0.012	1	Mature	No
o__Clostridiales;f__Unclassified;g__Unclassified	1.2770	0.5104	2.5018	0.012	1	Mature	No
[Clostridium]	-1.5784	0.6335	-2.4916	0.013	1	Immature	No
Lactobacillus	1.3226	0.5315	2.4884	0.013	1	Mature	No
f__Caulobacteraceae;g__Unclassified	1.8505	0.7494	2.4693	0.014	1	Mature	No
o__Bacillales;f__Unclassified;g__Unclassified	0.9025	0.3685	2.4490	0.014	1	Mature	No
f__Bacillaceae;g__Unclassified	0.9025	0.3685	2.4490	0.014	1	Mature	No
f__Micrococcaceae;g__Unclassified	0.9025	0.3685	2.4490	0.014	1	Mature	No
f__Piscirickettsiaceae;g__Unclassified	0.9025	0.3685	2.4490	0.014	1	Mature	No
c__SAR202;o__Unclassified;f__Unclassified;g__Unclassified	0.9025	0.3685	2.4490	0.014	1	Mature	No
o__[Pedosphaerales];f__Unclassified;g__Unclassified	0.9025	0.3685	2.4490	0.014	1	Mature	No
o__Chloroflexales;g__Unclassified;g__Unclassified	0.9025	0.3685	2.4490	0.014	1	Mature	No
f__Syntrophobacteraceae;g__Unclassified	0.9025	0.3685	2.4490	0.014	1	Mature	No
o__Solirubrobacterales;f__Unclassified;g__Unclassified	0.9025	0.3685	2.4490	0.014	1	Mature	No
Megasphaera	1.4838	0.6266	2.3681	0.018	1	Mature	No
Mitsuokella	-2.7968	1.1891	-2.3520	0.019	1	Immature	No
Baumannia	0.4051	0.1722	2.3519	0.019	1	Mature	No
Phaeobacter	2.4154	1.0317	2.3413	0.019	1	Mature	No
Phenylobacterium	0.9633	0.4128	2.3335	0.020	1	Mature	No
Enhydrobacter	0.9633	0.4128	2.3335	0.020	1	Mature	No
Porifericola	0.9633	0.4128	2.3335	0.020	1	Mature	No
Steroidobacter	0.9633	0.4128	2.3335	0.020	1	Mature	No
f__OM27;g__Unclassified	0.9633	0.4128	2.3335	0.020	1	Mature	No
HTCC	0.9633	0.4128	2.3335	0.020	1	Mature	No
Ralstonia	1.5923	0.6946	2.2923	0.022	1	Mature	No
Rahnella	-2.6676	1.1734	-2.2735	0.023	1	Immature	No
Campylobacter	1.8686	0.8300	2.2514	0.024	1	Mature	No
k__Bacteria;p__Proteobacteria;c__Betaproteobacteria;o__MND1;f__;g__Unclassified	1.0147	0.4511	2.2491	0.025	1	Mature	No
k__Bacteria;p__Cyanobacteria;c__ML635J-21;o__Unclassified;f__;g__Unclassified	1.0147	0.4511	2.2491	0.025	1	Mature	No
f__Conexibacteraceae;g__Unclassified	1.0147	0.4511	2.2491	0.025	1	Mature	No
f__Xenococcaceae;g__Unclassified	1.0147	0.4511	2.2491	0.025	1	Mature	No
Desulfobulbus	1.6957	0.7690	2.2051	0.027	1	Mature	No
Parabacteroides	0.4760	0.2172	2.1916	0.028	1	Mature	No
Kineococcus	1.0592	0.4848	2.1847	0.029	1	Mature	No

f__Paenibacillaceae;g__Unclassified	1.0592	0.4848	2.1847	0.029	1	Mature	No
c__Gemm-2;o__Unclassified;f__;g__Unclassified	1.0592	0.4848	2.1847	0.029	1	Mature	No
f__Rhodothermaceae;g__Unclassified	1.0592	0.4848	2.1847	0.029	1	Mature	No
Ammoniphilus	1.0592	0.4848	2.1847	0.029	1	Mature	No
B-42	1.0984	0.5148	2.1336	0.033	1	Mature	No
Anaerostipes	1.0984	0.5148	2.1336	0.033	1	Mature	No
f__Geodermatophilaceae;g__Unclassified	1.0984	0.5148	2.1336	0.033	1	Mature	No
Euzebya	1.4561	0.6871	2.1193	0.034	1	Mature	No
Actinomyces	1.5175	0.7195	2.1092	0.035	1	Mature	No
Rubricoccus	1.1335	0.5419	2.0919	0.036	1	Mature	No
c__Gitt-GS-136;o__Unclassified;f__Unclassified;g__Unclassified	1.1335	0.5419	2.0919	0.036	1	Mature	No
f__Planococcaceae;g__Unclassified	1.3603	0.6595	2.0625	0.039	1	Mature	No
Mycoplasma	1.8769	0.9103	2.0619	0.039	1	Mature	No
f__Rhizobiaceae;g__Unclassified	1.8532	0.8991	2.0611	0.039	1	Mature	No
Jiangella	1.1653	0.5665	2.0572	0.040	1	Mature	No
Swaminathania	1.1653	0.5665	2.0572	0.040	1	Mature	No
Pseudonocardia	1.1943	0.5890	2.0276	0.043	1	Mature	No
Chondromyces	1.1943	0.5890	2.0276	0.043	1	Mature	No
DA101	1.9534	0.9645	2.0253	0.043	1	Mature	No
Terracoccus	1.2210	0.6099	2.0021	0.045	1	Mature	No
o__Bacillales;g__Unclassified;g__Unclassified	1.2210	0.6099	2.0021	0.045	1	Mature	No
Geodermatophilus	1.2210	0.6099	2.0021	0.045	1	Mature	No
f__Actinosynnemataceae;g__Unclassified	1.2457	0.6292	1.9798	0.048	1	Mature	No
Afifella	1.2457	0.6292	1.9798	0.048	1	Mature	No
c__Betaproteobacteria;o__Unclassified;f__Unclassified;g__Unclassified	1.2689	0.6411	1.9794	0.048	1	Mature	No
c__Gammaproteobacteria;o__Unclassified;f__Unclassified;g__Unclassified	1.2687	0.6472	1.9602	0.050	1	Mature	No
f__Hyphomicrobiaceae;g__Unclassified	1.2687	0.6472	1.9602	0.050	1	Mature	No
f__Erythrobacteraceae;g__Unclassified	1.2687	0.6472	1.9602	0.050	1	Mature	No

LFC, log fold-change estimated by ANCOM-BC; positive values indicate enrichment in the Mature stage relative to Immature. SE, standard error of the LFC estimate. W, Wald test statistic (LFC/SE). p-value, nominal p-value from the Wald test. q-value, p-value adjusted for multiple testing using the Benjamini–Hochberg false discovery rate (FDR) procedure. Enriched in, the grape developmental stage in which the taxon shows higher relative abundance based on the direction of the LFC. Significant, whether the taxon passed FDR correction at $q < 0.05$ (Yes) or was nominally significant but did not survive multiple testing correction (No). Unclassified or partially annotated taxa are shown with their lowest available taxonomic assignment. Brackets around a genus name indicate provisional taxonomic designations in the original Greengenes taxonomy.

Table S4. Alpha diversity indices of the 16S rRNA-based GBM microbiota, excluding *Wolbachia*-associated sequences.

Sample	Observed features	Shannon	Pielou's Evenness	Chao1	Fisher	Simpson
Immature 1	426	4.62	0.53	580.92	87.95	0.92
Immature 2	247	5.01	0.63	256.97	41.57	0.93
Immature 3	309	5.80	0.70	316.73	53.98	0.95
Immature 4	260	4.62	0.58	281.69	44.54	0.88
Mature 1	311	4.93	0.60	327.27	52.30	0.85
Mature 2	391	6.45	0.75	393.40	75.62	0.97
Mature 3	576	6.70	0.73	582.22	122.33	0.96

Table S5. Quality control statistics of 18S rRNA gene sequences of GBM gut-associated microbes from larvae fed on immature and mature grapes.

Sample-id	Input	Filtered	Percent of input passed filter	Denoised	Merged	Percent of input merged	Non-chimeric	Percent of input non-chimeric
Immature-1	176,492	160,341	90.85	159,162	156,152	88.48	149,188	84.53
Immature-2	171,995	155,786	90.58	154,944	153,033	88.98	149,622	86.99
Immature-3	175,528	160,085	91.2	159,288	157,284	89.61	154,139	87.81
Mature-1	177,805	161,698	90.94	161,462	160,772	90.42	156,033	87.76
Mature-2	182,611	166,483	91.17	166,236	165,783	90.78	161,978	88.7
Mature-3	170,528	155,821	91.38	155,663	155,305	91.07	152,959	89.7
Mature-4	170,959	155,501	90.96	155,194	154,532	90.39	150,147	87.83

Table S6. Differential abundance analysis of fungal taxa at the family level from GBM larvae fed on grapes at two developmental stages (Immature vs. Mature) using ANCOM-BC (Analysis of Compositions of Microbiomes with Bias Correction) implemented in QIIME 2. Only taxa with a p-value < 0.05 are shown.

Family	LFC	SE	W	p value	q value	Enriched in	Significant
Saccharomycodaceae	9.4353	0.2569	36.7256	2.85E-292	1.68E-291	Mature	Yes
LKM11	-7.1308	0.2490	-28.6355	2.43E-180	1.41E-178	Immature	Yes
Gromochytriaceae	-3.4807	0.1707	-20.3881	2.13E-92	1.22E-90	Immature	Yes
uncultured	-4.8726	0.2768	-17.6055	2.24E-69	1.25E-67	Immature	Yes
Metschnikowiaceae	5.9643	0.3653	16.3267	6.38E-60	3.51E-58	Mature	Yes
Aphelidea	-3.0775	0.2133	-14.4272	3.49E-47	1.89E-45	Immature	Yes
Saccharomycetaceae	7.4373	0.5357	13.8845	7.86E-44	4.17E-42	Mature	Yes
Rhizophydiaceae	-2.3748	0.1927	-12.3233	6.78E-35	3.53E-33	Immature	Yes
Incertae_Sedis	4.7538	0.3994	11.9036	1.13E-32	5.78E-31	Mature	Yes
Pichiaceae	7.5421	0.6517	11.5733	5.63E-31	2.81E-29	Mature	Yes
Incertae_Sedis	3.7216	0.3263	11.4073	3.85E-30	1.88E-28	Mature	Yes
Saccharomycopsidaceae	5.9964	0.5409	11.0853	1.48E-28	7.09E-27	Mature	Yes
c__Chytridiomycetes;o__Unclassified;f__Unclassified	-2.2275	0.2520	-8.8409	9.50E-19	4.46E-17	Immature	Yes
Diaporthales	3.4057	0.5154	6.6078	3.90E-11	1.79E-09	Mature	Yes
Peronosporomycetes	-4.6738	0.7566	-6.1774	6.52E-10	2.93E-08	Immature	Yes
o__Hypocreales;f__Unclassified	3.7543	0.6411	5.8564	4.73E-09	2.08E-07	Mature	Yes
Mucoraceae	-1.7261	0.3008	-5.7394	9.50E-09	4.09E-07	Immature	Yes
Incertae_Sedis	-4.7405	0.9199	-5.1533	2.56E-07	1.07E-05	Immature	Yes
Mortierellaceae	-2.0693	0.4477	-4.6219	3.80E-06	1.56E-04	Immature	Yes
uncultured	-2.2490	0.5129	-4.3850	1.16E-05	4.64E-04	Immature	Yes
Cladosporiaceae	-1.9451	0.4675	-4.1605	3.18E-05	0.0012	Immature	Yes
Incertae_Sedis	-1.9572	0.4740	-4.1289	3.65E-05	0.0014	Immature	Yes
Trichocomaceae	-2.5707	0.6263	-4.1049	4.05E-05	0.0015	Immature	Yes
Nectriaceae	-2.8501	0.7386	-3.8589	1.14E-04	0.0041	Immature	Yes
Plectosphaerellaceae	-2.1138	0.5683	-3.7195	2.00E-04	0.0070	Immature	Yes
c__Dothideomycetes;o__Unclassified;f__Unclassified	2.6286	0.7108	3.6982	2.17E-04	0.0074	Mature	Yes
Chaetomiaceae	-1.1897	0.3824	-3.1109	0.0019	0.0597	Immature	No
Bulleribasidiaceae	-2.0923	0.6730	-3.1090	0.0019	0.0597	Immature	No
Aspergillaceae	2.4698	0.8163	3.0257	0.0025	0.0744	Mature	No
Oikopleuridae	-1.2410	0.4190	-2.9620	0.0031	0.0886	Immature	No
Pleosporaceae	-2.7302	0.9482	-2.8794	0.0040	0.1116	Immature	No
Lichtheimiaceae	-1.0545	0.4206	-2.5071	0.0122	0.3286	Immature	No
Sordariaceae	-1.0545	0.4206	-2.5071	0.0122	0.3286	Immature	No
Rhynchogastremataceae	-1.1335	0.4933	-2.2977	0.0216	0.5394	Immature	No
uncultured	-1.2410	0.5506	-2.2538	0.0242	0.5810	Immature	No
Dipodascaceae	1.2161	0.5706	2.1312	0.0331	0.7607	Mature	No
Sclerotiniaceae	1.6146	0.7797	2.0707	0.0384	0.8444	Mature	No

LFC, log fold-change estimated by ANCOM-BC; positive values indicate enrichment in the Mature stage relative to Immature. SE, standard error of the LFC estimate. W, Wald test statistic (LFC/SE). p-value, nominal p-value from the Wald test. q-value, p-value adjusted for multiple testing using the Benjamini–Hochberg false discovery rate (FDR) procedure. Enriched in, the grape

developmental stage in which the taxon shows higher relative abundance based on the direction of the LFC. Significant, whether the taxon passed FDR correction at $q < 0.05$ (Yes) or was nominally significant but did not survive multiple testing correction (No). Unclassified or partially annotated taxa are shown with their lowest available taxonomic assignment. Brackets around a family name indicate provisional taxonomic designations in the original Silva taxonomy.

Table S7. Differential abundance analysis of fungal taxa at the genus level from GBM larvae fed on grapes at two developmental stages (Immature vs. Mature) using ANCOM-BC (Analysis of Compositions of Microbiomes with Bias Correction) implemented in QIIME 2. Only taxa with a p-value < 0.05 are shown.

Genus	LFC	SE	W	p value	q value	Enriched in	Significant
Hanseniaspora	10.5841	0.2496	42.3970	0	0	Mature	Yes
Saturnispora	8.5938	0.3550	24.2048	1.98E-129	1.49E-127	Mature	Yes
LKM11	-5.9820	0.2589	-23.1094	3.72E-118	2.75E-116	Immature	Yes
Issatchenkia	9.1797	0.4303	21.3311	5.85E-101	4.27E-99	Mature	Yes
Clavispora-Candida_clade	7.1131	0.3835	18.5493	8.26E-77	5.95E-75	Mature	Yes
Starmerella-Candida_clade	5.9026	0.4069	14.5077	1.08E-47	7.69E-46	Mature	Yes
Tilletiopsis	4.8704	0.3397	14.3373	1.28E-46	8.96E-45	Mature	Yes
uncultured	-3.7239	0.2733	-13.6278	2.74E-42	1.89E-40	Immature	Yes
Saccharomycopsis	7.1452	0.5501	12.9895	1.40E-38	9.55E-37	Mature	Yes
uncultured	-2.2615	0.1949	-11.6026	4.00E-31	2.68E-29	Immature	Yes
Pichia	8.1495	0.7063	11.5390	8.39E-31	5.54E-29	Mature	Yes
Martiniozyma	3.8065	0.3651	10.4250	1.91E-25	1.24E-23	Mature	Yes
uncultured	-1.8916	0.1832	-10.3268	5.33E-25	3.41E-23	Immature	Yes
Kurtzmaniella-Candida_clade	4.1242	0.4517	9.1300	6.85E-20	4.32E-18	Mature	Yes
f__Diaporthales;g__Unclassified	4.5545	0.5254	8.6689	4.36E-18	2.71E-16	Mature	Yes
o__Hypocreales;f__Unclassified;g__Unclassified	4.9031	0.6148	7.9755	1.52E-15	9.26E-14	Mature	Yes
Rhizophydium	-1.2260	0.1665	-7.3616	1.82E-13	1.09E-11	Immature	Yes
Ogataea-Candida_clade	3.2005	0.5745	5.5711	2.53E-08	1.49E-06	Mature	Yes
c__Dothideomycetes;o__Unclassified;f__Unclassifie d;g__Unclassified	3.7773	0.7114	5.3100	1.10E-07	6.36E-06	Mature	Yes
c__Chytridiomycetes;o__Unclassified;f__Unclassified;g[illegible] ied	-1.0787	0.2481	-4.3475	1.38E-05	7.85E-04	Immature	Yes
Yarrowia	2.3649	0.5769	4.0991	4.15E-05	0.0023	Mature	Yes
Paramicrosporidium	-3.5918	0.9319	-3.8544	1.16E-04	0.0064	Immature	Yes
Zygoascus-Candida_clade	1.4786	0.4206	3.5151	4.40E-04	0.0237	Mature	Yes
Sclerotinia	2.7634	0.7886	3.5041	4.58E-04	0.0243	Mature	Yes
Malassezia	1.3484	0.3871	3.4832	4.95E-04	0.0258	Mature	Yes
o__Saccharomycetales;f__Unclassified;g__Unclassifi ed	1.3803	0.3981	3.4675	5.25E-04	0.0268	Mature	Yes
f__Aspergillaceae;g__Unclassified	3.7334	1.1095	3.3649	7.66E-04	0.0383	Mature	Yes
o__Helotiales;g__Unclassified;g__Unclassified	0.9030	0.2870	3.1456	0.0017	0.0812	Mature	No
f__Pezizaceae;g__Unclassified	1.5800	0.5115	3.0887	0.0020	0.0965	Mature	No
Kazachstania-Candida_clade	1.9159	0.6267	3.0572	0.0022	0.1050	Mature	No
Dactylella	0.9587	0.3223	2.9745	0.0029	0.1350	Mature	No
Boeremia	3.2978	1.1300	2.9183	0.0035	0.1584	Mature	No
Fusarium	-1.7013	0.7457	-2.2814	0.0225	0.9910	Immature	No
LKM15	0.9030	0.4071	2.2180	0.0266	1	Mature	No
Thermomyces	-1.3515	0.6112	-2.2114	0.0270	1	Immature	No
f__Peronosporomycetes;g__Unclassified	-1.6031	0.7258	-2.2088	0.0272	1	Immature	No
uncultured	1.3291	0.6042	2.2000	0.0278	1	Mature	No
Helotiales	1.0762	0.5005	2.1502	0.0315	1	Mature	No
uncultured	-1.1002	0.5156	-2.1338	0.0329	1	Immature	No
Hortaea	1.3894	0.6534	2.1263	0.0335	1	Mature	No
Mortierella	-0.9206	0.4486	-2.0522	0.0401	1	Immature	No
Haliphthoros	-2.5104	1.2291	-2.0425	0.0411	1	Immature	No
o__Onygenales;f__Unclassified;g__Unclassified	1.2334	0.6244	1.9752	0.0482	1	Mature	No

LFC, log fold-change estimated by ANCOM-BC; positive values indicate enrichment in the Mature stage relative to Immature. SE, standard error of the LFC estimate. W, Wald test statistic (LFC/SE). p-value, nominal p-value from the Wald test. q-value, p-value adjusted for multiple testing using the Benjamini–Hochberg false discovery rate (FDR) procedure. Enriched in, the grape developmental stage in which the taxon shows higher relative abundance based on the direction of the LFC. Significant, whether the taxon passed FDR correction at $q < 0.05$ (Yes) or was nominally significant but did not survive multiple testing correction (No). Unclassified or partially annotated taxa are shown with their lowest available taxonomic assignment. Brackets around a genus name indicate provisional taxonomic designations in the original Silva taxonomy.

Table S8. SRA run accessions and basic metadata for 16S/18S rRNA sequencing of GBM larval guts.

Accession (SRA run)	Marker / Type	Sample description	Bases
SRX19899753	18S rRNA-seq (fungi)	Gut fungal communities of GBM larvae fed on immature grapes — replicate 3	77.6M
SRX19899752	18S rRNA-seq (fungi)	Gut fungal communities of GBM larvae fed on immature grapes — replicate 2	75.9M
SRX19899751	18S rRNA-seq (fungi)	Gut fungal communities of GBM larvae fed on immature grapes — replicate 1	78.1M
SRX19899750	16S rRNA-seq (bacteria)	Gut bacterial communities of GBM larvae fed on mature grapes — replicate 3	92.8M
SRX19899749	16S rRNA-seq (bacteria)	Gut bacterial communities of GBM larvae fed on mature grapes — replicate 2	94.0M
SRX19899748	16S rRNA-seq (bacteria)	Gut bacterial communities of GBM larvae fed on mature grapes — replicate 1	93.7M
SRX19899747	16S rRNA-seq (bacteria)	Gut bacterial communities of GBM larvae fed on immature grapes — replicate 4	85.8M
SRX19899746	16S rRNA-seq (bacteria)	Gut bacterial communities of GBM larvae fed on immature grapes — replicate 3	89.0M
SRX19899745	18S rRNA-seq (fungi)	Gut fungal communities of GBM larvae fed on mature grapes — replicate 4	75.2M
SRX19899744	18S rRNA-seq (fungi)	Gut fungal communities of GBM larvae fed on mature grapes — replicate 3	75.2M
SRX19899743	18S rRNA-seq (fungi)	Gut fungal communities of GBM larvae fed on mature grapes — replicate 2	80.5M
SRX19899742	18S rRNA-seq (fungi)	Gut fungal communities of GBM larvae fed on mature grapes — replicate 1	78.6M
SRX19899741	16S rRNA-seq (bacteria)	Gut bacterial communities of GBM larvae fed on immature grapes — replicate 2	91.6M
SRX19899740	16S rRNA-seq (bacteria)	Gut bacterial communities of GBM larvae fed on immature grapes — replicate 1	86.2M

Supplementary figures:

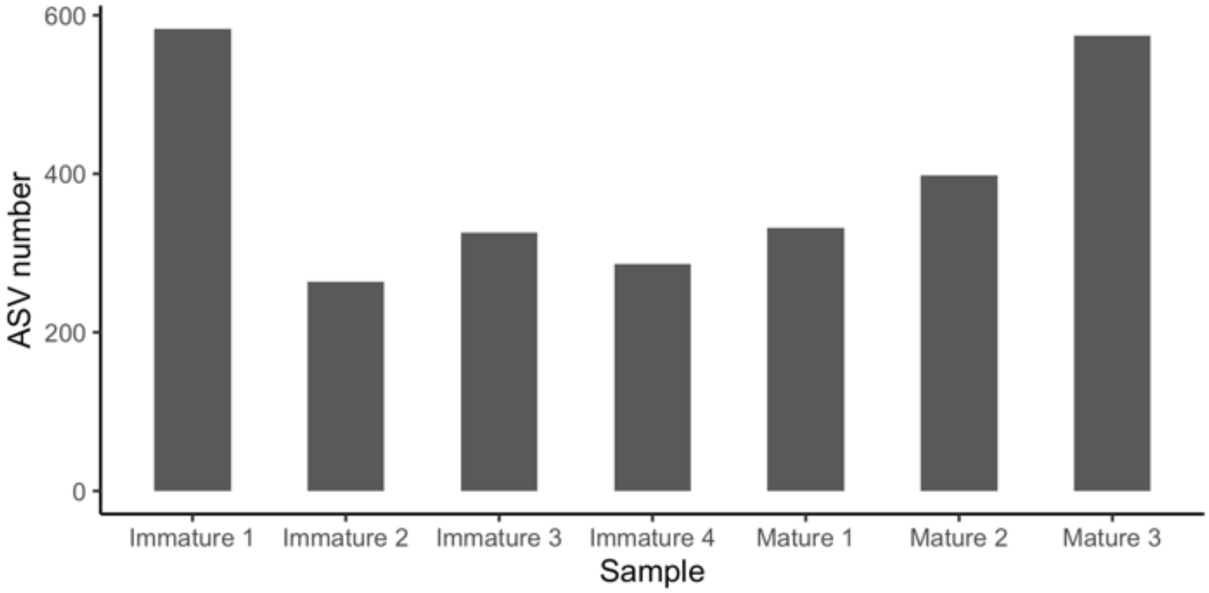

Figure S1. Number of bacterial taxonomic units (ASV) obtained per sample.

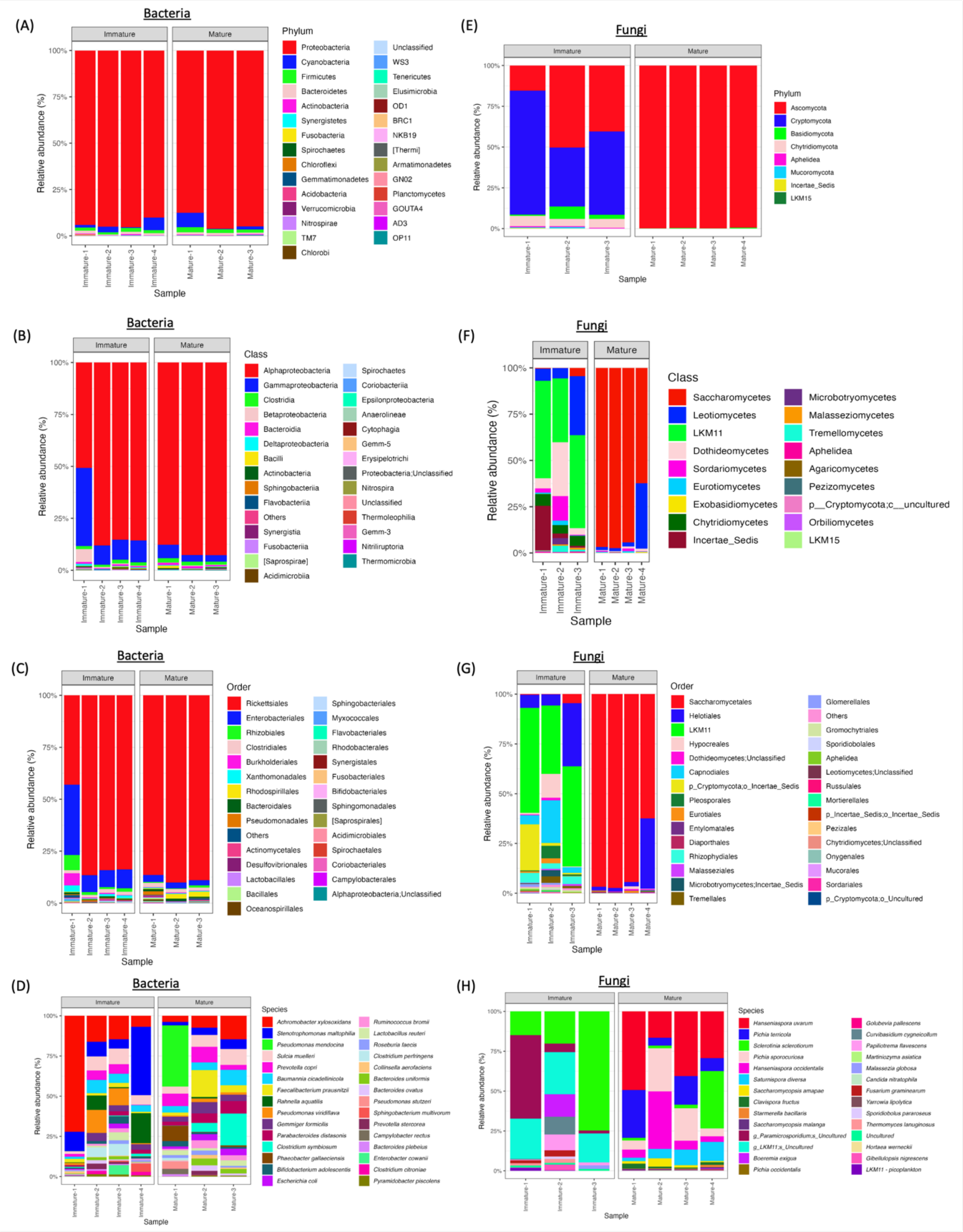

Figure S2. Relative abundance (%) of the gut microbial communities in *P. viteana* larvae across all samples. (A-D) Bacterial communities. (E-H) Fugal communities. Taxa levels are (A & E) phylum, (B & F) class, (C & G) order, and (D & H) putative species level after removing unclassified features. Taxa below the top 30-

abundance cut-off are represented as ‘Others’. ‘Unclassified’ represents taxa that have not been assigned. The *Wolbachia* reads were omitted from the bacterial species plot because their disproportionate abundance would have skewed the relative-abundance scale and masked the composition and dynamics of the remaining community members.

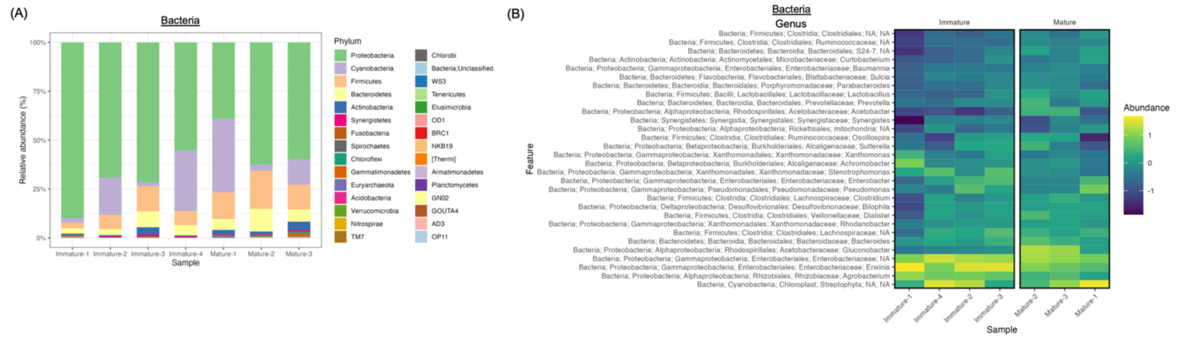

Figure S3. Relative abundance of the gut bacterial communities in *P. viteana* larvae across all samples after removing the *Wolbachia* sequences. (A) Relative abundances at the phylum level, (B) Heatmap of relative abundances at the genus level. ‘Unclassified’ represents taxa that have not been assigned.

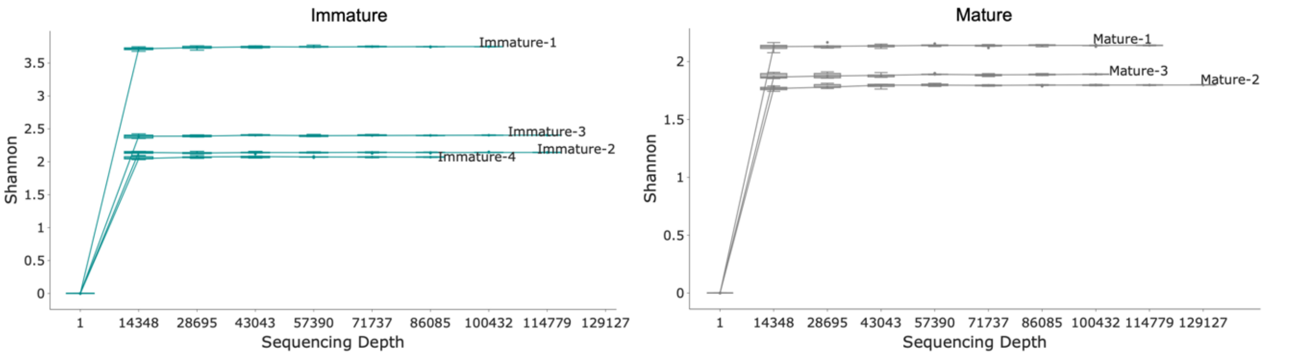

Figure S4. Rarefaction curves showing species richness of GBM gut bacterial communities from larvae fed on immature and mature grapes.

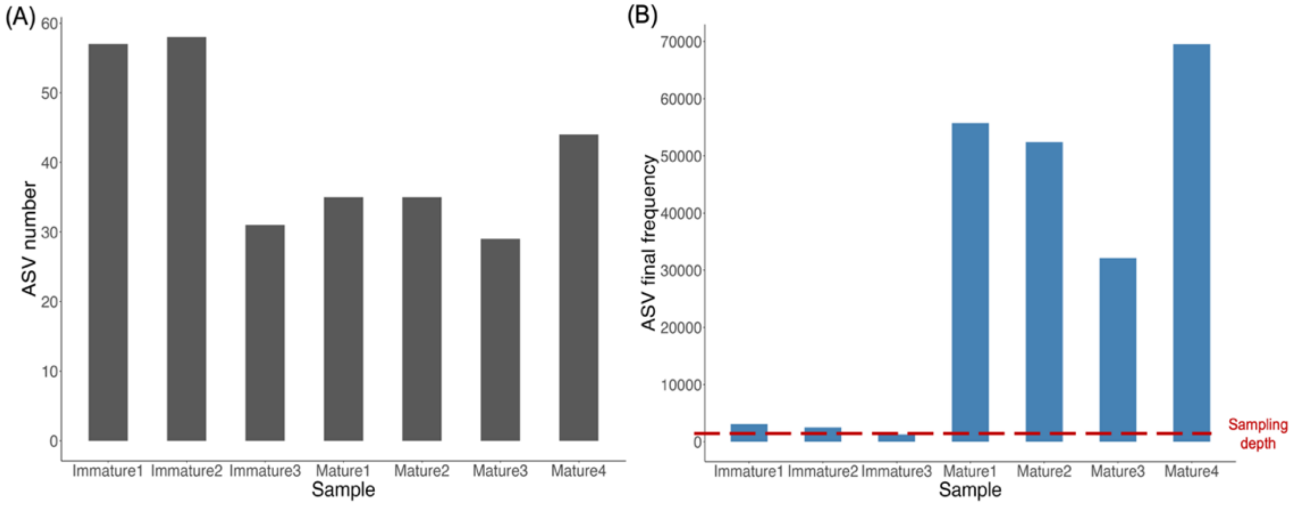

Figure S5. Fungal amplicon sequence variants (ASVs) conserved per sample after taxonomic filtering. (A) Number of conserved features (ASVs), (B) Frequency of ASVs. The red-dashed line indicates the hypothetical sampling depth needed to normalize the samples.
